# Associate Principal Investigators and the HEAL-COVID trial: good for trainees, good for trials

**DOI:** 10.1186/s13063-024-07936-x

**Published:** 2024-01-27

**Authors:** Joseph Newman, Philip Wild, HEAL-COVID Collaboration, Charlotte Summers, Mark Toshner

**Affiliations:** 1 Victor Phillip Dahdaleh Heart and Lung Research Institute, University of Cambridge, Cambridge, UK; 2 Royal Papworth Hospital, Cambridge, UK

**Keywords:** Associate Principal Investigators, Clinical trials, Training, Patient recruitment, COVID-19

## Abstract

**Background:**

The NIHR’s Associate Principal Investigator (API) Scheme in the United Kingdom was expanded nationally in 2020 with the aim of training clinicians to become Principal Investigators for clinical research in the future. The HEAL-COVID adaptive platform trial is an urgent public health study registered with the API Scheme. Within eighteen months of opening, the trial had recruited almost 1200 patients with over 100 active sites. Here we describe our experiences of APIs working on the trial with two broad objectives. Firstly, we aim to explore through qualitative methods the impact that the scheme has had on the APIs’ professional development. Secondly, we aim to quantify the impact that the APIs have had on the recruitment of patients into the trial.

**Methods:**

The professional backgrounds of the APIs are described from data from their application forms to the scheme. The HEAL-COVID API Network is described from records of the monthly meetings. The APIs’ experiences are reviewed from data from the NIHR exit surveys at 6 months and from a reflective practice exercise at the final network meeting. Data of patient recruitment to HEAL-COVID was analysed for centres with and without APIs via a multivariate analysis.

**Results:**

Forty-two APIs were registered with the HEAL-COVID trial with a diversity of backgrounds in terms of gender, country, profession, grade and specialty. Eleven monthly network meetings took place with the dual objectives of facilitating trial activity and providing educational content. Fourteen APIs completed the NIHR survey with all reporting Good Clinical Practice completion, local promotional activity of the trial, patient recruitment and support from their respective PI. Sites with at least one API recruited over 3.5 times more patients than sites without an API (medians 4 vs 14.5, *p* < 0.05), independent of factors including type of hospital or number of inpatient beds.

**Discussion:**

This study adds to the growing literature that the NIHR’s API Scheme is effective in meeting its objectives in providing research training to clinicians, thus building a workforce of future clinical researchers. Moreover, data from the HEAL-COVID trial shows that sites with an API are associated with higher recruitment. Overall, registering a trial with the API Scheme not only trains future clinical researchers, but it is also likely to increase the number of patients recruited (amongst other benefits), increasing the efficiency of trials and improving access for patients.

**Supplementary Information:**

The online version contains supplementary material available at 10.1186/s13063-024-07936-x.

## Background

The National Institute of Health and Care Research (NIHR) was established in the UK in 2006 under the government’s health research strategy to support clinical research within the National Health Service (NHS) [1]. It is the largest funder of health and social care research in the UK and works in collaboration with cross-sector partners. Following the success of a pilot scheme within surgical clinical trials that commenced in February 2019, the NIHR opened the Associate Principal Investigator (API) Scheme to more additional clinical specialties in 2020 [2]. The programme has grown significantly since this time, in large part due to nationally prioritised Urgent Public Health COVID-19 clinical trials such as the RECOVERY trial, to reach over 1000 APIs by April 2022 [3].

The API Scheme is a nationwide flagship programme aiming to provide clinicians without prior research experience with the skills needed to support research delivery. It is endorsed by the Royal Colleges and the General Medical Council [4]. Through an apprenticeship model, the programme aims to develop doctors, nurses and other staff in clinical practice into the Principal Investigators (PIs) of the future. Following an application process, APIs are mentored by the site PI for a specific NIHR CRN portfolio study for a period of 6 months. In addition, they receive online training and need to complete a checklist of activities.

Helping Alleviate the Longer-term consequences of COVID-19 (HEAL-COVID) is an adaptive, randomised, phase 3, open-label, multi-centre, superiority platform trial (Clinical Trials.gov: NCT04801940) [5]. The primary objective is to determine whether interventions in the post-acute phase of COVID-19 improve longer-term mortality and morbidity outcomes. HEAL-COVID was registered with the NIHR API Scheme and engagement of APIs was identified as a key priority early on. The first API application was received on 8th May 2021. The first site opened to recruitment on 7th May 2021 with the first patient recruited on 19th May 2021. By October 2022 almost 1200 participants had been randomised into the trial with a peak of 106 sites active across the UK.

As a relatively recent development in clinical trial mechanics, there are limited data regarding both the scheme’s impact on the professional development of APIs and the impact that of the contributions made by APIs on the delivery of clinical trials. Here we report on our experiences of the API Scheme within the context of HEAL-COVID, drawing upon multiple sources of quantitative and qualitative data.

The aims of this work are to describe the experience of APIs involved in HEAL-COVID and the impact on professional development. In addition to the impact of the scheme on APIs, we also investigated the relationship between APIs and participant recruitment.

## Methods

### API cohort description

The HEAL-COVID API cohort is described from data recorded on NIHR scheme application forms, including gender, profession, specialty, grade, and country.

### API professional development

The outcomes relating to the professional development of the HEAL-COVID APIs are drawn from two sources. Firstly, all APIs are invited to complete a feedback questionnaire by the NIHR in the sixth month of the scheme covering topics including trial activities, training, reflections and future career plans. These anonymised binary and free-text responses were analysed with descriptive statistics and qualitatively via iterative coding and emergent thematic analysis. Verbatim quotes from the free-text responses were coded and clustered into themes when there was independent repetition of the same concept from different APIs, without investigators having a priori pre-conception of themes and regardless of whether the scheme was viewed positively or negatively. Completion of the survey is not mandatory so data missingness is expected. Anonymised data were provided by the NIHR API team for use in this study.

Secondly, in the final HEAL-COVID API Network meeting of the 2021/22 academic year, the APIs were asked to reflect on their experiences of participation. Responses were captured digitally via the Vevox polling app [6]. Word clouds were created illustrating all reflections, with themes most frequently reported represented in larger font. The anonymous responses provided by APIs were shared for the purposes of publication, and the data were captured without any demographic or identifiable elements.

### HEAL-COVID API national network

The HEAL-COVID API Network is described from data recorded at each monthly meeting by the author (JN) who convened this, including the number of sessions and format.

### API impact on patient recruitment

As a proxy measure for the impact that APIs’ had on trial delivery, the absolute numbers of patients recruited to HEAL-COVID are compared for sites that registered at least one API compared with those that did not register an API. Due to the non-normal distribution of these data, medians are compared nonparametrically using the Mann–Whitney *U* test.

Having shown a significant association between patient recruitment and registration of an API at a site, multiple linear regression was undertaken to model other possible independent variables that may impact recruitment or be associated with the presence of a site API.

Four additional variables were included in the model:

- Duration of time (days) the site had been open to recruitment (continuous)
- Type of hospital: district general hospital or teaching/tertiary hospital (categorical)
- Presence of an acute admissions service or not (categorical)
- Number of inpatient beds (continuous)

These variables were selected as ones that could feasibly impact on both patient recruitment and/or presence of an API. The data were either collected as part of the trial (e.g. duration of site being open) or were obtained from publicly available open resources (e.g. acute admissions service) including NHS Trust websites.

The terms for the model with unweighted variables were:

$$Recruitment = \beta 0 + \beta1^{*}\!API + \beta2^{*}\!time + \beta3^{*}\!hospital + \beta4^{*}\!acute + \beta5^{*}\!beds$$

Reference levels for categorical variables were set at zero/baseline (no API, general hospital, no acute service) to which comparisons were made. The parameter estimates (coefficients) were calculated with a confidence interval set at 95%, representing how much higher the average recruitment per site was for each variable, taking into account the other variables. The goodness of fit of the model was measured by multiple *R* and *R*^2^. Multi-colinearity was calculated for each variable with covariance shown in a correlation matrix heatmap; this was performed by the least squares method in GraphPad Prism (Version 9.3.1).

All data are anonymised and non-identifiable, and the authors were given permission by the NIHR to use it for this purpose.

## Results

### API cohort description

**Fig. 1:**
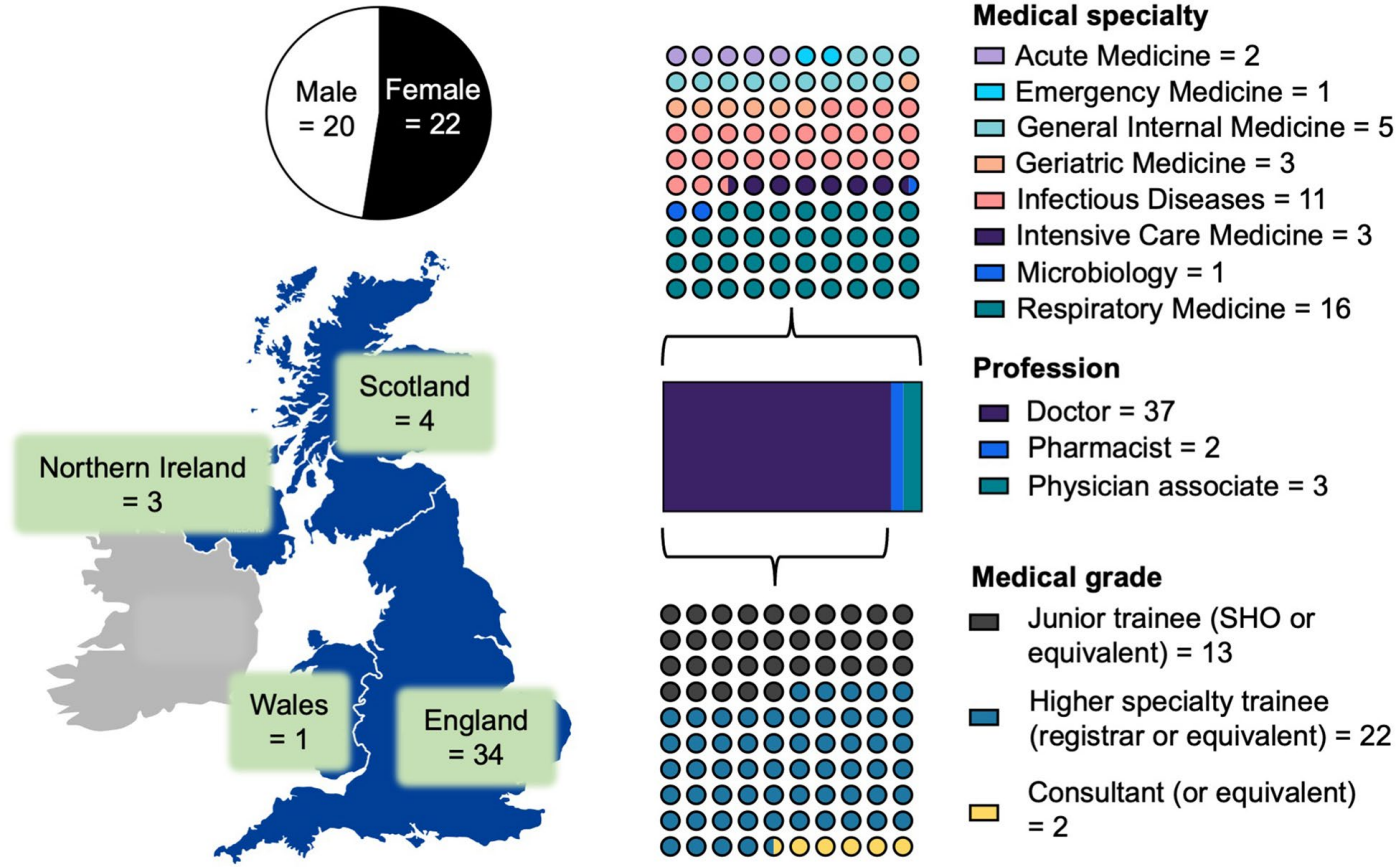
An infographic of the demographics of APIs in the HEAL-COVID trial including gender, country, profession, grade and medical specialty

The demographic data of the APIs is shown in Fig. 1. Of the 42 APIs registered with HEAL-COVID, 22 are female. APIs were located across all four nations of the United Kingdom, with a predominance in England (34/42). The professional backgrounds were doctor (37/42), pharmacist (2/42) and physician associate (3/42). The APIs practiced clinically in eight different medical specialties. The most highly represented were respiratory medicine (15/42), infectious diseases (11/42), and general internal medicine (3/42). The doctors’ grades included those at Junior Trainee (Senior House Officer) or equivalent (13/37), Higher Specialty Trainee (Registrar) or equivalent (22/37), and Consultant or equivalent (2/37). All 42 of the APIs who registered for the scheme with HEAL-COVID went on to successfully complete it as per the NIHR requirements.

### API professional development

The NIHR online exit survey was completed by 14 out of 26 APIs who had completed the 6-month scheme at the point of analysis (Supplement 1a). Of these, all had completed Good Clinical Practice (GCP) training [7], and all had undertaken activity to promote the trial at their local site. All 14 APIs felt adequately supported in the role and reported meeting their PI daily (1/14), weekly (11/14) and monthly (2/14). The self-reported number of patients the APIs each recruited were: 1 to 5 (5/14), 6 to 10 (3/14), 11 to 20 (5/14) and more than 20 (1/14). All the APIs reported that their experiences had encouraged them to become more engaged with research once they had completed the scheme.

The extended free-text responses from the NIHR survey were reviewed qualitatively with emergent coding and subsequent thematic analysis. Eleven significant themes emerged:

- Learning and training
- Teamwork
- Types of activity undertaken
- Future career plans
- Patient benefit
- Leadership skills and responsibility
- Engagement with the HEAL-COVID API Network
- Little prior research experience
- Enjoyable experience
- Recommend scheme to others
- Clinical research embedded in routine care

Further information on this qualitative analysis, including verbatim quotes from free-text responses is available in Supplement 1b.

**Fig. 2:**
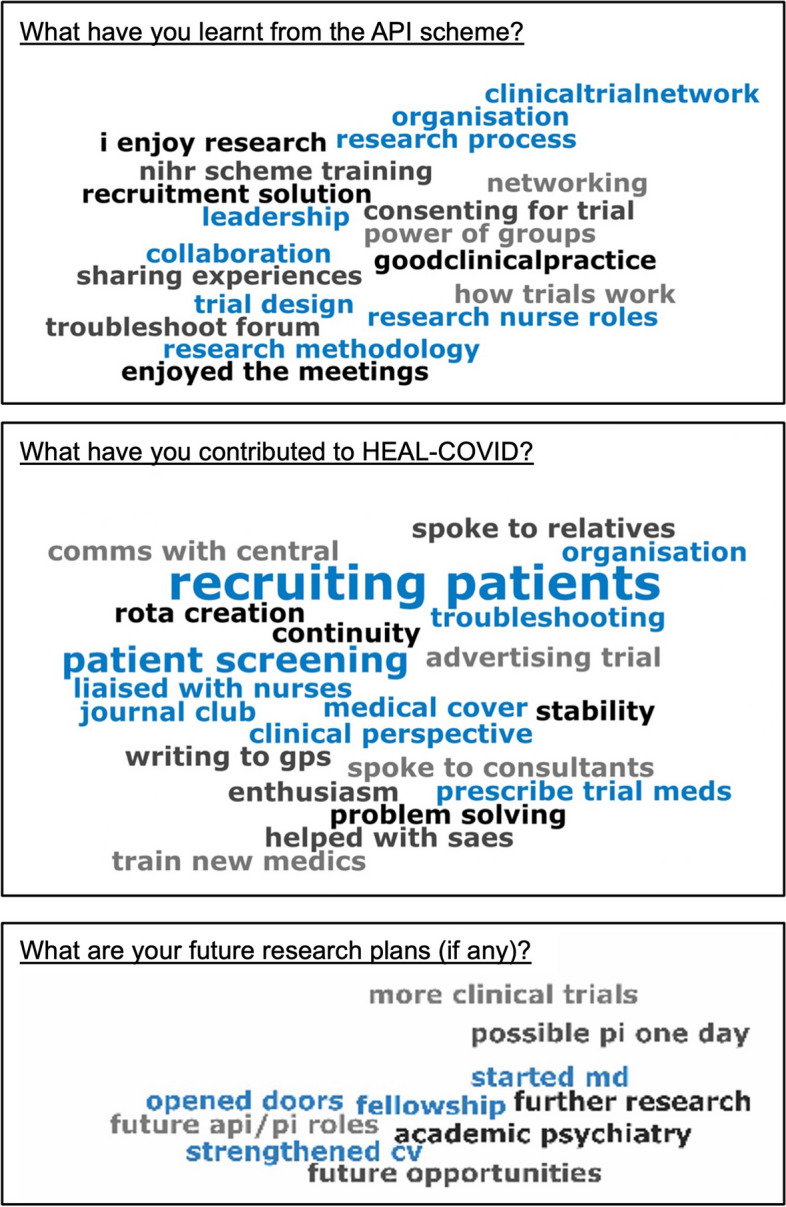
Word clouds of anonymous reflections from APIs in response to three questions. The larger the font size, the more frequent the response

The wordclouds in Fig. 2 illustrate the reflective responses from the APIs regarding what they learnt from and contributed to the trial and any subsequent future research career plans. The most frequent response regarding contributions to the running of HEAL-COVID was that of “recruiting patients”.

### HEAL-COVID API national network

There were 11 HEAL-COVID API Network meetings between October 2021 and August 2022. Meetings were typically an hour in length and conducted via Zoom videoconferencing [8], with invitations sent to all APIs registered for the trial. The meeting format consisted of two halves to the meeting: discussion of the trial and an educational session. Trial updates were communicated regarding participant numbers, protocol amendments, social media, sharing resources and any other news. There was then an ‘open forum’ troubleshooting discussion in which APIs could share experiences and help formulate strategies to implement the trial using others’ successful local experiences. Finally, the educational sessions included guest speakers (three) and presentations by APIs (five) regarding an aspect of the trial, a journal club paper review or other related topic. The discussion from the network meetings was fed back to the Chief Investigators and trial team. This included snapshot polls when rapid structured feedback was needed to help inform trial progress and amendments.

### API impact on patient recruitment

**Fig. 3:**
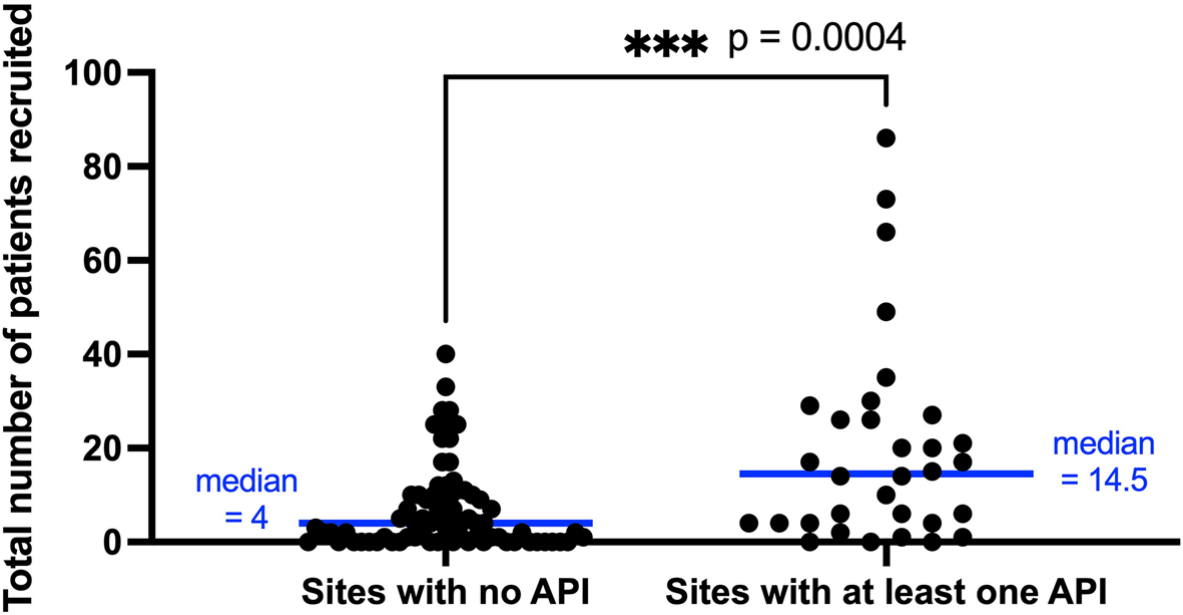
A comparison of the total number of patients recruited to HEAL-COVID for sites without an API vs sites which have had at least one API

Patient recruitment data was censored on 13th September 2022. Sites that have never had an API registered for HEAL-COVID recruited a median of four patients compared to a median of 14.5 patients recruited at sites that have had at least one API (*p* = 0.004) (Fig. 3).

Multiple linear regression was used to test if the presence of one or more registered APIs, time (in days) open to recruitment, type of hospital, presence of an acute admissions service and number of inpatient beds significantly predicts the total number of patients recruited to HEAL-COVID by site. 109 complete data sets representing each open site within the HEAL-COVID trial were used.

We found that only the presence of an API (*β* = 13.4, *p* < 0.001) and the duration of time open to recruitment (*β* = 0.0481, *p* =  < 0.001) significantly predicted patient recruitment (Table 1).

**Table 1:** Parameter estimates (coefficients) with 95% confidence intervals for each of five variables

Variable	Variable label	Estimate (coefficient)	95% CI	*P* value	*P* value summary
β1	Any API(s)?	13.4	7.22 to 19.5	< 0.001	***
β2	Duration open?	0.0481	0.0202 to 0.0761	< 0.001	***
β3	Teaching/specialist hospital?	− 1.62	− 8.28 to 5.04	0.63	ns
β4	Acute admissions?	− 0.028	− 10.2 to 10.2	> 0.99	ns
β5	Number of inpatient beds?	0.00258	− 0.00909 to 0.0143	0.66	ns

^***^signifies statistical signifance with a *P* value ≤ 0.001

The multiple regression model showed a moderate goodness of fit with multiple *R* of 0.471 and *R*^2^ of 0.222 for actual versus predicted recruitment using the model (Supplement 2a).

There was moderate collinearity for the variables β3 (type of hospital; 0.348) and β5 (number of beds; 0.371) but the other three independent variables show no significant collinearity (Supplement 2b). The covariance between these two parameters was − 0.54, with no significant interaction between the other independent variables (Supplement 2c).

## Discussion

This study shows that sites with a registered API recruited on average 3.6 times as many patients into HEAL-COVID, independent of other variables. APIs from a range of professional backgrounds reported receiving effective research training which is likely to positively impact on their future careers.

The HEAL-COVID cohort of APIs shows an equal gender balance, spread across appropriate medical specialties for a COVID-19 trial and a range of grades within the medical training structure. 12% of trainees were from non-medical backgrounds. This shows diversity in gender and professional background.

The results from the survey and the reflective practice network session are concordant with previous qualitative research about the API Scheme [9] and suggested that the APIs’ perceptions were that the programme provided them with education to help them develop their research skills, they felt that they contributed to the trial, and also that they are more likely to pursue a career including at least some clinical research in the future. The HEAL-COVID API Network was an opportunity to share good practice, troubleshoot, educate, incentivise and enthuse the APIs. The importance of forming peer support and educational networks outside of the API scheme seemed to have a positive impact on clinical trial recruitment as shown by a study within a trial (SWAT) regarding trainee PIs in the WHiTE 88 COPAL trial (ISRCTN No: 15606075) that investigated perioperative antibiotic use in hip fracture surgery [10]. The educational component of the API Scheme cohesively guides and incentivises involvement in a trial and thus patient recruitment.

The reported advantages of the scheme to APIs include structured education through network meetings and GCP training, access to online learning material, spin-off quality improvement projects, certification from an accredited scheme, career development opportunities, and recognition in publications as part of the HEAL-COVID Collaboration. Involvement with the scheme often represented their first opportunity to undertake impactful research and appeared to be acting as a stepping stone to future research opportunities. APIs reported a cycle in which they would encourage, recruit, train and hand over to colleagues to provide a continued API presence thus contributing to improving hospital and wider NHS clinical research culture [11]. For trials that are purposefully designed to be embedded in routine clinical care, such as HEAL-COVID, these benefits can be realised by the API with little additional burden placed on-site clinicians who are best placed within a hospital to screen potential participants [12]. This entrenchment of research into routine care and the concept that clinical research should be an “inherent part of training and professional development” have previously been highlighted by the RECOVERY trial [13].

The successes of the API scheme will increase the likelihood that APIs will be PIs in the future, as supported by our data and that of others [9]. Indeed, 98% of graduates from the API scheme affiliated with the RECOVERY trial remained engaged in research [4].

The advantages of the scheme with regard to clinical trial delivery include an additional member of the research team, who is often a frontline healthcare worker from a range of relevant clinical backgrounds. APIs are well placed for case-finding and as a self-selected cohort are often enthusiastic about recruiting trial participants. APIs bring fresh perspectives, along with local knowledge and understanding, and an ability to promote the trial to colleagues. The APIs’ continued presence dedicated to a single trial helps trial sustainability and deliverability at a site, which is particularly relevant for pandemic studies with fluctuations in patient and staff numbers. These “ears on the ground” can feedback successes or barriers within the delivery of a trial to the central team via the network or directly.

The additional benefits that APIs bring to research teams are likely to contribute to our finding that sites with an API recruit more patients. The multiple linear regression has shown that the presence of one of more APIs at a site at any point during the trial is associated with a higher total number of participants recruited per site, regardless of the type of hospital service or the inpatient bed capacity. Whether a trial site has an API or not is also independent of these factors. APIs are not more or less likely to be registered at a general hospital compared to a teaching hospital, for example, and this category of hospital has no influence on recruitment.

The duration of time that a clinical trial has been open at an individual site is also associated with the total number of participants recruited at that site, but with a very small coefficient. Whilst it may be expected that the longer a site has been open, the greater the opportunity for participant recruitment, this contributed little to the model as most sites in HEAL-COVID opened very near the start of the overall trial, with some subsequently recruiting large numbers of participants and others less so.

Whilst there was collinearity between the type of hospital and inpatient bed numbers, this is neither surprising (teaching hospitals tend to be larger) nor of significance as neither factor is shown to be associated with recruitment.

The implications of enhanced patient recruitment are significant—trials that recruit more quickly are reduced in length, cost, and time to clinical implementation of the findings—all the more important in an Urgent Public Health clinical trial such as HEAL-COVID.

The Sunflower randomised control trial (ISRCTN No: 10378861), studying symptomatic gallbladder disease, found that trainee surgeons contributed to patient recruitment alongside consultants and research nurses [9]. This effect was greater for acute than elective admissions, which is most analogous to the HEAL-COVID population who were acutely hospitalised. The acute setting is perhaps where APIs can add most value. The Sunflower API study included only more senior trainee, not all of whom were registered as APIs, and there was no control group (sites without trainees/APIs). It is unclear whether trainees impacted the overall recruitment of participants to the trial, or whether the same patients would have been recruited by other investigators.

One valid criticism of the API scheme is the voluntary nature, which is likely to introduce disadvantage related to the capacity to contribute to what is additional work on top of clinical duties. A lack of reimbursement and recognition within job plans is a widespread problem for clinical staff contributing to research, and despite the very positive accounts of the scheme from participants, there will be a bias in the group of people participating in the API programme.

Our study is limited by the modest number of APIs (42) and an NIHR survey completion rate of only 54% (14/26) at the point of analysis. Whilst there is moderate goodness of fit of the regression model, it clearly does not account for all factors that influence participant recruitment. Further work should be undertaken to understand and possibly optimise these as yet unknown contributing factors.

Research into the perspectives of and impacts on PIs in relation to the scheme is important to evaluate the premise of workload reduction. Similarly, qualitative research into participant perceptions on their interactions with APIs as junior researchers should be conducted. It is also pertinent to understand the barriers to API registration at sites that have not registered an API. The key question of impact on recruitment is best addressed by a prospective, ideally randomised trial.

Overall, this mixed-methods study demonstrates the synergy between the API Scheme and HEAL-COVID trial. In training junior clinical researchers to be the PIs of the future, their contribution to the running of the trial has increased the number of participants recruited at their sites. Chief Investigators should consider registering their clinical trials with the NIHR’s API Scheme, and seek to engage with APIs as they are a proven efficacious resource in research now and in the future.

### Supplementary Information

**Additional file 1:** **Supplement 1.** a). Summary of quantitative data from NIHR online exit survey sent to APIs at 6 months. b) Summary of 11 themes, summaries of the emergent codes with example verbatim quotes from extended qualitative responses from NIHR online exit surveys from APIs at 6 months. **Supplement 2.** a) A plot of actual vs predicted recruitment using the multiple regression model. b) Multicolinearity showing R2 for each of the five variables. c) Heat map matrix of parameter covariance. **Supplement 3.** The HEAL-COVID Collaboration.

## Data Availability

The datasets used and/or analysed during the current study are available from the corresponding author on reasonable request.
