# Supplementary material for: Associate Principal Investigators and the HEAL-COVID trial: good for trainees, good for trials
Source: Trials. 2024 Jan 27;25:90. doi: 10.1186/s13063-024-07936-x (PMC10821510; doi:10.1186/s13063-024-07936-x)
Supplement: Supplementary file 1 — Additional file 1: Supplement 1. a). Summary of quantitative data from NIHR online exit survey sent to APIs at 6 months. b) Summary of 11 themes, summaries of the emergent codes with example verbatim quotes from extended qualitative responses from NIHR online exit surveys from APIs at 6 months. Supplement 2. a) A plot of actual vs predicted recruitment using the multiple regression model. b) Multicolinearity showing R2 for each of the five variables. c) Heat map matrix of parameter covariance. Supplement 3. The HEAL-COVID Collaboration. [file 13063_2024_7936_MOESM1_ESM.docx]

**Supplement 1.**

1. **Summary of quantitative data from NIHR online exit survey sent to APIs at six-months**

| n = 14 |  |  |  |  |
| --- | --- | --- | --- | --- |
| **Did you complete GCP training?** | Yes 14 (100%) |  |  |  |
| **How many patients have you recruited since registering with the scheme?** | 1-5 5 (36%) | 6-10 3 (21%) | 11-20 5 (36%) | >20 1 (7%) |
| **Have you undertaken any activity to promote the trial at your centre (eg distributing information to colleagues, speaking at departmental meeting)?** | Yes 14 (100%) |  |  |  |
| **How often do you have contact with the local PI?** | Daily 1 (7%) | Weekly 11 (79%) | Monthly 2 (14%) |  |
| **Do you feel adequately supported in this role?** | Yes 14 (100%) |  |  |  |
| **Have your experiences as an API encouraged you to become more engaged with research once you complete the scheme?** | Yes 14 (100%) |  |  |  |

1. **Summary of 11 themes, summaries of the emergent codes with example verbatim quotes from extended qualitative responses from NIHR online exit surveys from APIs at six-months**

| **Theme** | **Summary of emergent codes from free text responses** | **Example quotes from free text responses** |
| --- | --- | --- |
| **Learning and training** | APIs report that significant amounts of learning took place through hands-on experience and via a steep learning curve they developed decision-making and problem-solving skills. | - allowed me to learn so much about the process of research - pick up so much about research - very quick learning curve - eye opening experience - witnessed first hand - trouble-shoot issues |
| **Teamwork** | APIs report feeling part of the research team alongside the PI and experienced research nurses in a supportive and supervised role. | - part of the research team - Working closely with the PI and experienced research nurses - supervised environment - support from my PI - Mentoring to support - so many people working as a team - amazing to see what teamwork can achieve |
| **Types of activity undertaken** | APIs report actively assisting with set-up, delivery, recruitment, promotion of the trial to colleagues and liaison with the trial’s central team. | - setup, delivery, and recruitment - making decisions on recruiting patients and problem solving - front facing recruitment - work 'behind the scenes' - distribute information amongst clinical teams to improve engagement - recruit patients - liased with the central team |
| **Future career plans** | APIs report feeling inspired to continue involvement in clinical research or academia, actively seeking other opportunities to develop this aspect of their career. | - inspired me to become more involved in more multicentre studies - now continue throughout my career to try to continue with getting involved with research - advice on how to continue to be involved in research - look forward to the future with better research understanding - think more about a research career |
| **Patient benefit** | APIs report they recognised that their small contributions help advance evidence-based treatments and care for patients on a large scale. | - small contributions could help advance the evidence-based science of treating patients with the right treatments - the impact novel research can have on patient care - how it can benefit so many people - play my part in helping patients recover |
| **Leadership skills and responsibility** | APIs report developing management skills, gaining confidence to lead a team, and taking responsibility under supervision. | - Leadership and management skills - more confident in leading - leadership role within in the team - taking a leading role - having responsibility |
| **Engagement with the HEAL-COVID API Network** | APIs report learning from and connecting with colleagues across the UK which was enjoyable and useful. | - Engagement, networking and learning with colleagues from across the UK through HEAL COVID API group - being supported by teams throughout the UK and the central team coordinating it all. - gained connections with other APIs across the country - most enjoyed networking with other APIs at the monthly meeting - monthly HEAL-COVID API meetings very useful - API support sessions were really useful |
| **Little prior research experience** | APIs report that they had minimal or no prior research experience. | - involved minimally with research prior to this role - having had limited experience in research prior to the scheme |
| **Enjoyable experience** | APIs report thoroughly enjoying the scheme and experience of working on the rial. | - Very enjoyable to be involved - thoroughly enjoyed this experience - thoroughly enjoyed my time on the API scheme |
| **Recommend scheme to others** | APIs report that they had or would, definitely and strongly, recommend the scheme to colleagues. | - definitely recommend to others - Recommended to a colleague - would recommend it to anyone - strongly recommend the scheme to others |
| **Clinical research embedded in routine care** | APIs report recognising their role in helping to embed clinical research into routine care without significant additional amounts of work and how such pragmatic research reflects real-world clinical practice. | - research happens "in the real world" - research in the clinical setting - in a busy hospital environment |

**Supplement 2.**

1. **A plot of actual vs predicted recruitment using the multiple regression model.**

1. **Multicolinearity showing R^2^ for each of the five variables.**

| **Variable** | **Variable label** | **R^2^ with other variables** |
| --- | --- | --- |
| β1 | Any API(s)? | 0.0981 |
| β2 | Duration open? | 0.0278 |
| β3 | Teaching/specialist hospital? | 0.348 |
| β4 | Acute admissions? | 0.0948 |
| β5 | Number of inpatient beds? | 0.371 |

1. **Heat map matrix of parameter covariance.**

**Supplement 3.**

**The HEAL-COVID Collaboration**

**Writing Committee for this manuscript**

Joseph Newman, Philip Wild, Mark Toshner, Charlotte Summers.

**Trial Management Group**

Charlotte Summers (Chief Investigator)

Mark Toshner (Lead Investigator)

Carol Gamble

Thomas Jaki

Martin Landray

Danny McAuley

Gisli Jenkins

J Kenneth Baillie

Elin Haf Davies

Duncan Richards

Melanie Calvert

Paul Wicks

Annemarie Docherty

Dyfrig Hughes

Jonathan Mantt

Peter Openshaw

**Trial Steering Committee**

James Chalmers (Chair)

Wei-Shen Lim

Katherine Sleeman

John Hurst

Matt Sydes

Frances Mair

**Independent Data and Safety Monitoring Committee**

Duncan Young (Chair)

Theodore Jack Iwashyna

Hannah Durrington

Siobhan Creanor

**Liverpool Clinical Trials Centre, University of Liverpool**

| Carrol Gamble |
| --- |
| Ashley Jones |
| Ashley Best |
| Efstathia Gkioni |
| Emma Bedson |
| Rachael Dagnall |
| Chloe Donohue |
| Sharon Kean |
| Jonathan Gibb |
| Anthony Shorrock  Linda Kane  Clare Jackson  Michaela Brown  Janet Harrison  Simon Winn  Michelle Girvan  Helen Hickey |

**Central clinical trial team (University of Cambridge / Cambridge University Hospitals NHS Foundation Trust)**

Charlotte Summers

Mark Toshner

Joseph Newman

Sarah Hewitt

Sarah Berry

Sara Stearn

Carrie Bayliss

Rachel Slade

Mary Kasanicki

Nicola Forber

Denise Pinto

**National Institute for Health and Care Research**

Anne Priest (NIHR CRN East)

Harley Bircher (NIHR CRN East)

Sallyanne Hurford (NIHR CRN East)

Bonnie Jackson (NIHR CRN East)

Nick Lemoine (NIHR CRN National)

Issy Baker (NIHR CRN National)

Ben Drew (NIHR CRN National)

Kate Fenton (NIHR NETSCC)

**Site teams**

| Aberdeen Royal Infirmary | Jamie Cooper | Principal Investigator |  |
| --- | --- | --- | --- |
| Aberdeen Royal Infirmary | Pauline Ganley |  |  |
| Aberdeen Royal Infirmary | Andrew Nicolson | Associate PI |  |
| Aberdeen Royal Infirmary | Robin Brittain-Long |  |  |
| Aberdeen Royal Infirmary | Jenny Noble |  |  |
| Aberdeen Royal Infirmary | Janice Irvine |  |  |
| Aberdeen Royal Infirmary | Robert Laing |  |  |
| Aberdeen Royal Infirmary | Lukman Hakeem |  |  |
| Aberdeen Royal Infirmary | Lynne Walker |  |  |
| Aberdeen Royal Infirmary | Roy Soiza |  |  |
| Addenbrooke's Hospital | Jonathan Fuld | Principal Investigator |  |
| Addenbrooke's Hospital | Joanne La Verne Sanchez |  |  |
| Addenbrooke's Hospital | Jason Domingo |  |  |
| Addenbrooke's Hospital | Maria Vivien Mendoza |  |  |
| Addenbrooke's Hospital | Elena Robisco Diaz |  |  |
| Addenbrooke's Hospital | Hoi Ping Mok |  |  |
| Addenbrooke's Hospital | Kirsty Harvey |  |  |
| Addenbrooke's Hospital | Ciro Pasquale |  |  |
| Addenbrooke's Hospital | Rachel Bousfield | Associate PI |  |
| Addenbrooke's Hospital | Suny Coscione | Associate PI |  |
| Addenbrooke's Hospital | Karen Brookes |  |  |
| Addenbrooke's Hospital | Evgenia Kourampa |  |  |
| Addenbrooke's Hospital | Rose Swain |  |  |
| Addenbrooke's Hospital | Thanos Mamarelis | Associate PI |  |
| Addenbrooke's Hospital | Eleanor Lim | Associate PI |  |
| Addenbrooke's Hospital | Jimmy Agato |  |  |
| Altnagelvin Area Hospital | Martin Kelly | Principal Investigator |  |
| Altnagelvin Area Hospital | Kathryn Ferguson |  |  |
| Altnagelvin Area Hospital | Declan Mcclintock |  |  |
| Altnagelvin Area Hospital | Breda Robinson |  |  |
| Altnagelvin Area Hospital | Declan Garvey |  |  |
| Altnagelvin Area Hospital | Valerie Mortland |  |  |
| Bedford Hospital | Timothy Chapman | Principal Investigator |  |
| Bedford Hospital | Ruby Fronda |  |  |
| Bedford Hospital | Dinesh Bagmane |  |  |
| Bedford Hospital | Laura Ylquimiche Melly |  |  |
| Bedford Hospital | Areeb Mian |  |  |
| Bedford Hospital | Areeb A Mian |  |  |
| Bedford Hospital | Melchizedek Penacerrada |  |  |
| Bedford Hospital | Mel Penacerrada |  |  |
| Bedford Hospital | Joshua Sarella |  |  |
| Bedford Hospital | Wura-Ola Makinde |  |  |
| Bedford Hospital | Yuuki Yanagisawa |  |  |
| Bedford Hospital | Uzoamaka Keke |  |  |
| Belfast City Hospital | Danny McAuley | Principal Investigator |  |
| Belfast City Hospital | Roisin Stone |  |  |
| Belfast City Hospital | Danielle Dawson |  |  |
| Belfast City Hospital | Jonathan Stewart |  |  |
| Belfast City Hospital | Brian Wells |  |  |
| Belfast City Hospital | Karan Smyth |  |  |
| Belfast City Hospital | Alexandra Usher-Rea |  |  |
| Belfast City Hospital | Siobhan Moor |  |  |
| Belfast City Hospital | Samantha Murphy |  |  |
| Belfast City Hospital | Suzanne Tauro |  |  |
| Belfast City Hospital | Abitha Balakrishnan Nair |  |  |
| Belfast City Hospital | Ronan Doherty | Associate PI |  |
| Belfast City Hospital | Fred McElwaine | Associate PI |  |
| Blackpool Victoria Hospital | David Mcghee | Principal Investigator |  |
| Blackpool Victoria Hospital | Melanie Caswell |  |  |
| Blackpool Victoria Hospital | Sarah Melling |  |  |
| Blackpool Victoria Hospital | Leonie Benham |  |  |
| Blackpool Victoria Hospital | Ella Riedel |  |  |
| Blackpool Victoria Hospital | Stephanie White |  |  |
| Blackpool Victoria Hospital | Scott Warden |  |  |
| Blackpool Victoria Hospital | Emma Ward |  |  |
| Blackpool Victoria Hospital | Lisa Elawamy |  |  |
| Blackpool Victoria Hospital | Alexander Bulcock |  |  |
| Blackpool Victoria Hospital | Anastasia Pseftinakou |  |  |
| Bradford Royal Infirmary | Paul Whitaker | Principal Investigator |  |
| Bradford Royal Infirmary | Jackie Todd |  |  |
| Bradford Royal Infirmary | Bridget Tandy |  |  |
| Bradford Royal Infirmary | Matt Aldred |  |  |
| Bristol Royal Infirmary | Emma Stratton | Principal Investigator |  |
| Bristol Royal Infirmary | Lauren Morgan | Associate PI |  |
| Bristol Royal Infirmary | Liz Mccullagh |  |  |
| Bristol Royal Infirmary | Rebecca Houlihan |  |  |
| Bristol Royal Infirmary | Toni Farmery |  |  |
| Bristol Royal Infirmary | Joanna Willis |  |  |
| Bristol Royal Infirmary | Laura Beacham |  |  |
| Bristol Royal Infirmary | Karen Bobruk |  |  |
| Bristol Royal Infirmary | Brenda Chivima |  |  |
| Bristol Royal Infirmary | Heather Hudson |  |  |
| Bristol Royal Infirmary | Elysia Gower |  |  |
| Bristol Royal Infirmary | Victoria Haile |  |  |
| Bristol Royal Infirmary | Rosina Jarvis |  |  |
| Bristol Royal Infirmary | Wendy Chiu |  |  |
| Bristol Royal Infirmary | Mary Trevelyan |  |  |
| Bristol Royal Infirmary | Miriam Thake | Associate PI |  |
| Bristol Royal Infirmary | Samuel Brooks |  |  |
| Bristol Royal Infirmary | Alice Lagnado |  |  |
| Bristol Royal Infirmary | Zoe Woodward |  |  |
| Bristol Royal Infirmary | Jordan Mayer |  |  |
| Bristol Royal Infirmary | Ella Schofield |  |  |
| Bristol Royal Infirmary | Thomas Parry |  |  |
| Bristol Royal Infirmary | Molly Flint |  |  |
| Bristol Royal Infirmary | Abdul Muqsith Naha Kizakkiniyakath |  |  |
| Bristol Royal Infirmary | Christopher Stewart | Associate PI |  |
| Bristol Royal Infirmary | Victoria Roberts | |  |
| Bristol Royal Infirmary | Thomas Mayo |  |  |
| Bristol Royal Infirmary | Tara Nandwani |  |  |
| Bristol Royal Infirmary | Jasmine Chingono |  |  |
| Bristol Royal Infirmary | Carla Swanson-Low |  |  |
| Bristol Royal Infirmary | Alexandra Tate |  |  |
| Bristol Royal Infirmary | Caroline Woodman | Associate PI |  |
| Bristol Royal Infirmary | Abigail Cannon |  |  |
| Bristol Royal Infirmary | Jodie Servante |  |  |
| Bristol Royal Infirmary | Cameron Blair | Associate PI |  |
| Bristol Royal Infirmary | Rebecca Davies | Associate PI |  |
| Bronglais General Hospital | Maria Hobrok | Principal Investigator |  |
| Bronglais General Hospital | Ronda Loosley |  |  |
| Bronglais General Hospital | Heather Mcguinness |  |  |
| Bronglais General Hospital | Syed Raza |  |  |
| Bronglais General Hospital | Helen Tench |  |  |
| Bronglais General Hospital | Rebecca Wolf-Roberts |  |  |
| Bronglais General Hospital | Maddie Mathews |  |  |
| Bronglais General Hospital | William Wolf |  |  |
| Bronglais General Hospital | Angharad Eburne |  |  |
| Bronglais General Hospital | Ceclilie Klementsen |  |  |
| Bronglais General Hospital | Kaled Hatashe |  |  |
| Broomfield Hospital | Abi Moore | Principal Investigator |  |
| Broomfield Hospital | Temp Hold |  |  |
| Broomfield Hospital | Amanda Lyle |  |  |
| Broomfield Hospital | Sharon Reid |  |  |
| Broomfield Hospital | Emma Cannon |  |  |
| Broomfield Hospital | Iman Mohamed Mohamed |  |  |
| Broomfield Hospital | Jack Mulholland |  |  |
| Calderdale Royal Hospital | Anneka Biswas | Principal Investigator |  |
| Calderdale Royal Hospital | Matthew Robinson |  |  |
| Calderdale Royal Hospital | Lee-Ann Bayo |  |  |
| Calderdale Royal Hospital | Tracy Wood |  |  |
| Calderdale Royal Hospital | Steven Thomas |  |  |
| Calderdale Royal Hospital | Sarah Hoye |  |  |
| Calderdale Royal Hospital | Benjamin Callow | Associate PI |  |
| Calderdale Royal Hospital | Rehima Aslam |  |  |
| Calderdale Royal Hospital | Sophia Kim |  |  |
| Calderdale Royal Hospital | Basel Sakka |  |  |
| Calderdale Royal Hospital | Li Ji Ng |  |  |
| Calderdale Royal Hospital | Kathryn Smith |  |  |
| Calderdale Royal Hospital | Charlotte Spencer |  |  |
| Calderdale Royal Hospital | Trishala Raj |  |  |
| Calderdale Royal Hospital | Sanjay Kumar |  |  |
| Calderdale Royal Hospital | Eloise Smellie |  |  |
| Calderdale Royal Hospital | Sarah Hanley |  |  |
| Calderdale Royal Hospital | Sam Crickmore |  |  |
| Calderdale Royal Hospital | Hamza Naeem |  |  |
| Calderdale Royal Hospital | Des Ncube |  |  |
| Calderdale Royal Hospital | Madihah Nafeez |  |  |
| Calderdale Royal Hospital | Wajid Rashid |  |  |
| Calderdale Royal Hospital | Lisa Gledhill |  |  |
| Calderdale Royal Hospital | Kathleen Thompson |  |  |
| Calderdale Royal Hospital | Alison Abbott |  |  |
| Castle Hill Hospital | Nicholas Easom | Principal Investigator |  |
| Craigavon Area Hospital | Rory Convery | Principal Investigator |  |
| Craigavon Area Hospital | Michelle Kinnin |  |  |
| Craigavon Area Hospital | Denise Cosgrove |  |  |
| Craigavon Area Hospital | Denise Mcfarland |  |  |
| Craigavon Area Hospital | Sophie Hughes |  |  |
| Craigavon Area Hospital | Clare Mccullagh |  |  |
| Craigavon Area Hospital | Cormac Murtagh |  |  |
| Craigavon Area Hospital | Deborah Cullen |  |  |
| Cumberland Infirmary | Clive Graham | Principal Investigator |  |
| Darlington Memorial Hospital | Shafie Kamaruddin | Principal Investigator |  |
| Darlington Memorial Hospital | Julie Temple |  |  |
| Darlington Memorial Hospital | Susan Wadd |  |  |
| Darlington Memorial Hospital | Ellen Brown |  |  |
| Darlington Memorial Hospital | Dawn Egginton |  |  |
| Darlington Memorial Hospital | Sarah Mcauliffe |  |  |
| Darlington Memorial Hospital | Nicola Hewitson |  |  |
| Darlington Memorial Hospital | Kelly Postlethwaite |  |  |
| Darlington Memorial Hospital | Clare Brady |  |  |
| Darlington Memorial Hospital | Kimberly Stamp |  |  |
| Darlington Memorial Hospital | Amana Cowton |  |  |
| Darlington Memorial Hospital | Margaret Randall |  |  |
| Derriford Hospital | John Corcoran | Principal Investigator |  |
| Derriford Hospital | Glen Marsh | Associate PI |  |
| Derriford Hospital | Karen Friendship |  |  |
| Derriford Hospital | Memory Mwadeyi |  |  |
| Derriford Hospital | Hannah Newman |  |  |
| Derriford Hospital | Liam O'brien |  |  |
| Derriford Hospital | Ramy Mansour |  |  |
| Derriford Hospital | Chris Gordon |  |  |
| Derriford Hospital | Louise Jose |  |  |
| Derriford Hospital | Emily May |  |  |
| Derriford Hospital | Kelly Whitehorn |  |  |
| Diana, Princess of Wales Hospital | Obaid Khan | Principal Investigator |  |
| Diana, Princess of Wales Hospital | Rachael Stead |  |  |
| Diana, Princess of Wales Hospital | Sue Spencer |  |  |
| Diana, Princess of Wales Hospital | Kathy Dent |  |  |
| Diana, Princess of Wales Hospital | Rachel Pollard |  |  |
| Diana, Princess of Wales Hospital | Dinah Smith |  |  |
| Diana, Princess of Wales Hospital | Maria Newton |  |  |
| Diana, Princess of Wales Hospital | Alexandra Curran |  |  |
| Diana, Princess of Wales Hospital | Shivani Virpura |  |  |
| Diana, Princess of Wales Hospital | Albert Ang |  |  |
| Diana, Princess of Wales Hospital | Mohammed Hussein Abdi |  |  |
| Diana, Princess of Wales Hospital | Emily Pawley |  |  |
| Diana, Princess of Wales Hospital | Amira Bhaiji |  |  |
| Diana, Princess of Wales Hospital | Elaine Heeney |  |  |
| Diana, Princess of Wales Hospital | Teresa Behan |  |  |
| Diana, Princess of Wales Hospital | Ayesha Shah |  |  |
| Diana, Princess of Wales Hospital | Caroline Downing |  |  |
| Diana, Princess of Wales Hospital | Jack Sanders |  |  |
| Diana, Princess of Wales Hospital | Amelia Chalmers |  |  |
| Ealing Hospital | Padmasayee Papineni | Principal Investigator |  |
| Ealing Hospital | Sheena Quaid |  |  |
| Ealing Hospital | Sambasivarao Gurram |  |  |
| Ealing Hospital | George Hulston |  |  |
| Ealing Hospital | Alexander Vogt |  |  |
| Ealing Hospital | Ekaterina Watson |  |  |
| Epsom Hospital | Rebecca Macfarlane | Principal Investigator |  |
| Epsom Hospital | Victoria Taylor |  |  |
| Epsom Hospital | Annie Mathew |  |  |
| Epsom Hospital | Lisa Evans |  |  |
| Epsom Hospital | Nicole Blanco |  |  |
| Epsom Hospital | Grace Blows |  |  |
| Epsom Hospital | Manuel Rebolledo Romero |  |  |
| Epsom Hospital | Amy Matthews |  |  |
| Glasgow Royal Infirmary | Hannah Bayes | Principal Investigator |  |
| Glasgow Royal Infirmary | Jennifer Tait |  |  |
| Glasgow Royal Infirmary | Andrew Dougherty |  |  |
| Glasgow Royal Infirmary | Sharon Grant |  |  |
| Glasgow Royal Infirmary | Mark Smith |  |  |
| Glasgow Royal Infirmary | Erika Groth |  |  |
| Glasgow Royal Infirmary | Jaclyn Carberry |  |  |
| Glasgow Royal Infirmary | Hilary Wilson |  |  |
| Glasgow Royal Infirmary | Lucia Di Mascio |  |  |
| Glasgow Royal Infirmary | Rufus Turner |  |  |
| Glasgow Royal Infirmary | Erin Mcgarry | Associate PI |  |
| Glasgow Royal Infirmary | Visali Sivathavalal |  |  |
| Glasgow Royal Infirmary | Emma Johnson |  |  |
| Glasgow Royal Infirmary | Robert Sykes |  |  |
| Glasgow Royal Infirmary | Anne-Marie Munro |  |  |
| Glasgow Royal Infirmary | Siouxsie Mackenzie |  |  |
| Glasgow Royal Infirmary | Sofia Vanessa Silva |  |  |
| Glasgow Royal Infirmary | Jonathan Perkins |  |  |
| Glasgow Royal Infirmary | Renata Rebuitti |  |  |
| Glasgow Royal Infirmary | Dominic Rimmer |  |  |
| Glasgow Royal Infirmary | Gary Semple |  |  |
| Glasgow Royal Infirmary | Alexis Duncan |  |  |
| Glasgow Royal Infirmary | Alison Begg |  |  |
| Glasgow Royal Infirmary | Larissa Latif |  |  |
| Glasgow Royal Infirmary | Jocelyn Luveta |  |  |
| Glasgow Royal Infirmary | Erin Murphy |  |  |
| Glasgow Royal Infirmary | Laila Martin |  |  |
| Glasgow Royal Infirmary | Scott Griffiths |  |  |
| Glasgow Royal Infirmary | Megan Crawford |  |  |
| Glasgow Royal Infirmary | Annie Caldwell |  |  |
| Glasgow Royal Infirmary | Lynne Turner |  |  |
| Glasgow Royal Infirmary | Oluwatobi Adeagbo |  |  |
| Glasgow Royal Infirmary | Kirsty Hodgson |  |  |
| Glasgow Royal Infirmary | Kay Graham |  |  |
| Glasgow Royal Infirmary | Anne Milligan |  |  |
| Glasgow Royal Infirmary | Panagiotis Georgios Passias |  |  |
| Glasgow Royal Infirmary | Victoria Macleod |  |  |
| Glasgow Royal Infirmary | Michael Mccloskey |  |  |
| Glenfield Hospital | Rachael Evans | Principal Investigator |  |
| Glenfield Hospital | Jee Kim |  |  |
| Glenfield Hospital | Hamish Mcauley |  |  |
| Glenfield Hospital | Amanda Charalambou |  |  |
| Glenfield Hospital | Amelia Lea |  |  |
| Glenfield Hospital | Sarah Parker |  |  |
| Glenfield Hospital | Omer Elneima |  |  |
| Glenfield Hospital | Richard Russell |  |  |
| Glenfield Hospital | Sarah Diver |  |  |
| Glenfield Hospital | Joanne Finch |  |  |
| Glenfield Hospital | Iram Qureshi |  |  |
| Good Hope Hospital | Mohammad Saim | Principal Investigator |  |
| Good Hope Hospital | Bridget Hopkins |  |  |
| Good Hope Hospital | Heather Willis |  |  |
| Good Hope Hospital | Daniel Lenton |  |  |
| Good Hope Hospital | Kartikeya Misra |  |  |
| Good Hope Hospital | Humaira Pervaiz |  |  |
| Good Hope Hospital | Obaid Ullah |  |  |
| Good Hope Hospital | Kanza Arifeen |  |  |
| Good Hope Hospital | Abigail Roberts |  |  |
| Grange University Hospital | Prof Tamas Szakmany | Principal Investigator |  |
| Grange University Hospital | Gail Marshall |  |  |
| Grange University Hospital | Jemma Tuffney |  |  |
| Grange University Hospital | Sara Elizabeth Fairbairn |  |  |
| Grange University Hospital | Patricia James |  |  |
| Grange University Hospital | Abby Waters |  |  |
| Grange University Hospital | Claire Price |  |  |
| Grange University Hospital | Gemma Williams |  |  |
| Grange University Hospital | Jessica Nicholas |  |  |
| Guy's Hospital | Marlies Ostermann | Principal Investigator |  |
| Harefield Hospital | William Man | Principal Investigator |  |
| Harefield Hospital | VIBHA Teli |  |  |
| Harefield Hospital | Oliver Polgar |  |  |
| Harefield Hospital | Vivian Wu |  |  |
| Harefield Hospital | Jennifer Harvey |  |  |
| Heartlands Hospital | Gareth Walters | Principal Investigator |  |
| Heartlands Hospital | Mary Bellamy |  |  |
| Heartlands Hospital | Mirriam Sangombe |  |  |
| Heartlands Hospital | Emily Butler |  |  |
| Heartlands Hospital | Maria Lacson |  |  |
| Heartlands Hospital | Paul Ellis |  |  |
| Heartlands Hospital | Christopher Huntley |  |  |
| Heartlands Hospital | Patience Juru |  |  |
| Heartlands Hospital | Louise Wood |  |  |
| Heartlands Hospital | Faye Moore |  |  |
| Heartlands Hospital | Chris Reilly |  |  |
| Heartlands Hospital | Hollie Bancroft |  |  |
| Heartlands Hospital | Ed Birkhamshaw |  |  |
| Heartlands Hospital | Samson Sapele |  |  |
| Heartlands Hospital | Irfan SHAUKAT |  |  |
| Heartlands Hospital | Zakir Ullah |  |  |
| Heartlands Hospital | Mohamed Abdelwahab |  |  |
| Heartlands Hospital | Danai Papakonstantinou |  |  |
| Heartlands Hospital | William Osborne |  |  |
| Heartlands Hospital | Tammy Bellamy |  |  |
| Heartlands Hospital | Lauren Breslin |  |  |
| Hereford County Hospital | Ingrid Durand | Principal Investigator |  |
| Hereford County Hospital | Janine Birch |  |  |
| Hereford County Hospital | Samantha Turner |  |  |
| Hereford County Hospital | Kate Hammerton |  |  |
| Hereford County Hospital | Susan Anderson |  |  |
| Hereford County Hospital | Eleanor Andrews |  |  |
| Hereford County Hospital | Emma Collins |  |  |
| Hereford County Hospital | Hyeriju Gashau |  |  |
| Hereford County Hospital | Linda Moseley |  |  |
| Hereford County Hospital | Andrew Hedges |  |  |
| Hereford County Hospital | Jessica Annett |  |  |
| Hereford County Hospital | Serkan Cakir |  |  |
| Hereford County Hospital | Philip Ryan |  |  |
| Hereford County Hospital | Nimra Waheed |  |  |
| Hereford County Hospital | Sophie Gayle |  |  |
| Hereford County Hospital | Ann Bicknell |  |  |
| Hereford County Hospital | Jack Porteous |  |  |
| Hinchingbrooke Hospital | Rob Buttery | Principal Investigator |  |
| Hinchingbrooke Hospital | Roberta De Pretto |  |  |
| Hinchingbrooke Hospital | Victoria Christenssen |  |  |
| Hinchingbrooke Hospital | Eleanor Smith |  |  |
| Hinchingbrooke Hospital | Janet Taylor |  |  |
| Hinchingbrooke Hospital | Kathryn Leng |  |  |
| Hinchingbrooke Hospital | Beate Ebert |  |  |
| Hinchingbrooke Hospital | Alice Cole |  |  |
| Hinchingbrooke Hospital | Ping Coutts |  |  |
| Huddersfield Royal Hospital | Vincent Chau | Principal Investigator |  |
| Huddersfield Royal Hospital | Tracy Wood |  |  |
| Huddersfield Royal Hospital | Sarah Hoye |  |  |
| Huddersfield Royal Hospital | Lee-Ann Bayo |  |  |
| Huddersfield Royal Hospital | Matthew Robinson |  |  |
| Huddersfield Royal Hospital | Kathryn Smith |  |  |
| Huddersfield Royal Hospital | Simina Feier |  |  |
| Huddersfield Royal Hospital | Sam Dale |  |  |
| Huddersfield Royal Hospital | Karen Goodman |  |  |
| Huddersfield Royal Hospital | Lear Matapure |  |  |
| Huddersfield Royal Hospital | Naomi Chambers |  |  |
| Huddersfield Royal Hospital | Uzayr Akudi |  |  |
| Hull Royal Infirmary | Nicholas Easom | Principal Investigator |  |
| Hull Royal Infirmary | Katie Drury | Associate PI |  |
| Hull Royal Infirmary | Gemma Walker |  |  |
| Hull Royal Infirmary | Donna Norton |  |  |
| Hull Royal Infirmary | Dominic Sykes |  |  |
| Hull Royal Infirmary | Silmi Shahama |  |  |
| Hull Royal Infirmary | Rachael Ashworth |  |  |
| Hull Royal Infirmary | Ronke Williams |  |  |
| Hull Royal Infirmary | Adeleke Babatunde |  |  |
| James Cook University Hospital | David Chadwick | Principal Investigator |  |
| James Cook University Hospital | Marie Branch |  |  |
| James Cook University Hospital | Julie Potts |  |  |
| James Cook University Hospital | Pauline Lambert |  |  |
| James Cook University Hospital | Adele Szekeres |  |  |
| James Cook University Hospital | Waqar Ahmad |  |  |
| James Cook University Hospital | Irfan Iqbal Khan |  |  |
| James Cook University Hospital | John Mulcahy |  |  |
| James Cook University Hospital | Abigail Aboagye-Odei |  |  |
| James Cook University Hospital | Andrew Vaux |  |  |
| James Paget University Hospital | Jean Patrick | Principal Investigator |  |
| James Paget University Hospital | Katherine Mackintosh |  |  |
| James Paget University Hospital | Matthew Whelband |  |  |
| James Paget University Hospital | Jane Woods |  |  |
| James Paget University Hospital | Helen Sutherland |  |  |
| James Paget University Hospital | Elva Wilhelmsen |  |  |
| James Paget University Hospital | Wendy Harrison |  |  |
| James Paget University Hospital | Sally Dexter |  |  |
| James Paget University Hospital | Thrusha Jain |  |  |
| James Paget University Hospital | Amanda Ayers |  |  |
| James Paget University Hospital | Christian Hacon |  |  |
| James Paget University Hospital | Darylile Guledew |  |  |
| John Radcliffe Hospital | Brian Angus | Principal Investigator |  |
| John Radcliffe Hospital | Yolanda Warren |  |  |
| John Radcliffe Hospital | Maria Coates |  |  |
| John Radcliffe Hospital | Elizabeth Hadley |  |  |
| John Radcliffe Hospital | Vanessa Fenech |  |  |
| John Radcliffe Hospital | Musaiwale Kamfose |  |  |
| John Radcliffe Hospital | Laura Robledo |  |  |
| John Radcliffe Hospital | Ranoromanana Ionitiana |  |  |
| John Radcliffe Hospital | Angela Bloss |  |  |
| Leighton Hospital | Duncan Fullerton | Principal Investigator |  |
| Leighton Hospital | Katherine Pagett |  |  |
| Leighton Hospital | Claire Gabriel |  |  |
| Leighton Hospital | Tenifayo Adeyemo |  |  |
| Leighton Hospital | Deborah Maren |  |  |
| Leighton Hospital | Mandy Williams |  |  |
| Leighton Hospital | Christopher Brockelsby |  |  |
| Leighton Hospital | Sean Ward |  |  |
| Leighton Hospital | Georgia Morley |  |  |
| Leighton Hospital | Anna Burton | Associate PI |  |
| Lincoln County Hospital | Aqsa Aslam | Principal Investigator |  |
| Lincoln County Hospital | Grisha Furtado |  |  |
| Lincoln County Hospital | Muhammad Sabir |  |  |
| Lincoln County Hospital | Andrew Judd |  |  |
| Lincoln County Hospital | Gunjan Phalod |  |  |
| Lincoln County Hospital | Rebecca Spencer |  |  |
| Lincoln County Hospital | Susan Butler |  |  |
| Lincoln County Hospital | Silvia Tavares |  |  |
| Lincoln County Hospital | Nyan Tint |  |  |
| Lincoln County Hospital | Sarah Shephardson |  |  |
| Lincoln County Hospital | Kelly Hubbard |  |  |
| Lincoln County Hospital | Claire Hewitt |  |  |
| Lincoln County Hospital | Muhammad Umer Gill |  |  |
| Lincoln County Hospital | Mohamed Nassar |  |  |
| Lincoln County Hospital | Narcisse Ndoumbe |  |  |
| Lister Hospital | Alex Wilkinson | Principal Investigator |  |
| Lister Hospital | Katy-Jane Chick |  |  |
| Lister Hospital | Ashnish Sinha | Associate PI |  |
| Lister Hospital | Sheena Lim |  |  |
| Lister Hospital | Asmah Syed |  |  |
| Lister Hospital | Sura Dabbagh |  |  |
| Luton & Dunstable Hospital | Prashanth Shetty | Principal Investigator |  |
| Luton & Dunstable Hospital | Alex Francioni |  |  |
| Luton & Dunstable Hospital | Indika Perera |  |  |
| Luton & Dunstable Hospital | Naghma Riaz |  |  |
| Luton & Dunstable Hospital | Philippa Bakker |  |  |
| Luton & Dunstable Hospital | Margaret Louise Tate |  |  |
| Luton & Dunstable Hospital | Vasanth Sritharan |  |  |
| Luton & Dunstable Hospital | Arisa Reka |  |  |
| Luton & Dunstable Hospital | Iftikhar Nadeem |  |  |
| Mater Hospital | Danny McAuley | Principal Investigator |  |
| Mater Hospital | Siobhan Moor |  |  |
| Mater Hospital | Jonathan Stewart |  |  |
| Mater Hospital | Roisin Stone |  |  |
| Mater Hospital | Danielle Dawson |  |  |
| Mater Hospital | Brian Wells |  |  |
| Mater Hospital | Karan Smyth |  |  |
| Mater Hospital | Alexandra Usher-Rea |  |  |
| Mater Hospital | Samantha Murphy |  |  |
| Mater Hospital | Catherine Mcneill |  |  |
| Medway Maritime Hospital | Lisa Vincent-Smith | Principal Investigator |  |
| Medway Maritime Hospital | Laura Adams |  |  |
| Medway Maritime Hospital | Hiba Charaf |  |  |
| Medway Maritime Hospital | Mary Everett |  |  |
| Medway Maritime Hospital | Bosede Olaniyan |  |  |
| Medway Maritime Hospital | Younan Samuel |  |  |
| Medway Maritime Hospital | Dalhatu Yusuf |  |  |
| Monklands District General Hospital | Manish Patel | Principal Investigator |  |
| Monklands District General Hospital | Claire McGoldrick |  |  |
| Monklands District General Hospital | Lynn Glass |  |  |
| Monklands District General Hospital | Pauleen Grant |  |  |
| Monklands District General Hospital | Margaret McFadden |  |  |
| Monklands District General Hospital | Emma Lee |  |  |
| Monklands District General Hospital | Catriona Macrae |  |  |
| Monklands District General Hospital | Patrick Anstey |  |  |
| Monklands District General Hospital | Audrey McAlpine |  |  |
| Morriston Hospital | Ian Blyth | Principal Investigator |  |
| Morriston Hospital | Debra Evans |  |  |
| Morriston Hospital | Lorcan O'Connell | Associate PI |  |
| Morriston Hospital | Suzanne Richards |  |  |
| Morriston Hospital | Jenny Travers |  |  |
| Morriston Hospital | Abigail Holborow |  |  |
| Morriston Hospital | Caroline Davies |  |  |
| Morriston Hospital | Madeleine Shakeshaft |  |  |
| Musgrove Park Hospital | Ilinca Dragusin | Principal Investigator |  |
| Musgrove Park Hospital | Charmaine Shovelton |  |  |
| Musgrove Park Hospital | Rebecca Purnell |  |  |
| Musgrove Park Hospital | Chetan Pataki |  |  |
| Musgrove Park Hospital | Marius Vaida |  |  |
| Musgrove Park Hospital | Luke Rutter |  |  |
| Musgrove Park Hospital | Shilpa Nagendra |  |  |
| Musgrove Park Hospital | Anna Welbourn |  |  |
| Musgrove Park Hospital | Tim Richards |  |  |
| Musgrove Park Hospital | Gemma Chilcott |  |  |
| Musgrove Park Hospital | Laura Kyle |  |  |
| Newham University Hospital | Heinke Kunst | Principal Investigator |  |
| Newham University Hospital | Thomas Swaine |  |  |
| Newham University Hospital | Mylah Ramirez |  |  |
| Newham University Hospital | Ananna Rahman |  |  |
| Newham University Hospital | Catherine Heckman |  |  |
| Newham University Hospital | Malcolm Avari |  |  |
| Newham University Hospital | William Hann |  |  |
| Newham University Hospital | Neena Patel |  |  |
| Newham University Hospital | Maryam Omar |  |  |
| Newham University Hospital | Lawrence Langley |  |  |
| Ninewells Hospital | Mark Spears | Principal Investigator |  |
| Ninewells Hospital | Samera Mohammed |  |  |
| Ninewells Hospital | Africa Rhea Solstice |  |  |
| Ninewells Hospital | Arlene Shaw | Associate PI |  |
| North Manchester General Hospital | Andrew Ustianowski | Principal Investigator |  |
| North Manchester General Hospital | Gabriella Lindergard |  |  |
| North Manchester General Hospital | Sarah Holland |  |  |
| North Manchester General Hospital | Claire Fox |  |  |
| North Manchester General Hospital | Jan Flaherty |  |  |
| North Manchester General Hospital | Aliso Uriel |  |  |
| North Manchester General Hospital | Kathryn Ashton |  |  |
| North Manchester General Hospital | Samuel Hey |  |  |
| North Manchester General Hospital | Jacinta Guerin |  |  |
| North Manchester General Hospital | Bini George |  |  |
| North Manchester General Hospital | Thomas Scoones |  |  |
| North Manchester General Hospital | Kevin Kuriakose | Associate PI |  |
| North Tyneside General Hospital | Avinash Aujayeb | Principal Investigator |  |
| North Tyneside General Hospital | Abbie Tomlinson | Associate PI |  |
| North Tyneside General Hospital | Hayley Mckie |  |  |
| North Tyneside General Hospital | Jessica Bell |  |  |
| North Tyneside General Hospital | Jessica Reynolds |  |  |
| North Tyneside General Hospital | Angela Dawson |  |  |
| North Tyneside General Hospital | Tracy Smith |  |  |
| North Tyneside General Hospital | Stacey Short |  |  |
| North Tyneside General Hospital | Rachel Joseph |  |  |
| North Tyneside General Hospital | Sufyan Shakir | Associate PI |  |
| North Tyneside General Hospital | Gemma Mccafferty |  |  |
| North Tyneside General Hospital | Hannah Peggie |  |  |
| North Tyneside General Hospital | Maria Panteli |  |  |
| North Tyneside General Hospital | Joseph Kibbler |  |  |
| North Tyneside General Hospital | Lisa Gallagher |  |  |
| Northern General Hospital | Roger Thompson | Principal Investigator |  |
| Northern General Hospital | Claire Jarman |  |  |
| Northern General Hospital | Rosemary Kirk |  |  |
| Northern General Hospital | Joby Cole |  |  |
| Northern General Hospital | Alison Lye |  |  |
| Northern General Hospital | Helen Newell |  |  |
| Northern General Hospital | Kate Harrington |  |  |
| Northern General Hospital | Sara Jane Walker |  |  |
| Northern General Hospital | Lynne Smart |  |  |
| Northern General Hospital | Lorenza Onyinyechi Nwafor |  |  |
| Northern General Hospital | Sharon Megson |  |  |
| Northern General Hospital | Tom Newman |  |  |
| Northern General Hospital | Megan Plowright |  |  |
| Northwick Park Hospital | Ashley Whittington | Principal Investigator |  |
| Northwick Park Hospital | Meng-San Wu | Associate PI |  |
| Northwick Park Hospital | Ann Sturdy |  |  |
| Nottingham City Hospital | Stephen Ryder | Principal Investigator |  |
| Nottingham City Hospital | Jan Hallas |  |  |
| Nottingham City Hospital | Jack Squires |  |  |
| Nottingham City Hospital | Emma Kendall |  |  |
| Nottingham City Hospital | Sabrina Prosper |  |  |
| Nottingham City Hospital | Athanasios Nikolaidis |  |  |
| Nottingham City Hospital | Georgia Melia |  |  |
| Nottingham City Hospital | Nicola Benetti |  |  |
| Nottingham City Hospital | Chloe Khurana |  |  |
| Nottingham City Hospital | Marie Quinlan |  |  |
| Nottingham City Hospital | Catherine Dupont |  |  |
| Pilgrim Hospital | Pavlos Zafeiris | Principal Investigator |  |
| Pilgrim Hospital | Kimberley Netherton |  |  |
| Pilgrim Hospital | Andrew Judd |  |  |
| Pilgrim Hospital | Gunjan Phalod |  |  |
| Pilgrim Hospital | Helen Palmer |  |  |
| Pilgrim Hospital | Bryony Saint |  |  |
| Pilgrim Hospital | Kinga Szymiczek |  |  |
| Pilgrim Hospital | Malik Habib Asghar |  |  |
| Pilgrim Hospital | Victoria Azuamah |  |  |
| Pilgrim Hospital | Preethy Nath |  |  |
| Pilgrim Hospital | Kataryna Dos Santos |  |  |
| Pinderfields General Hospital | Muthu Thirumaran | Principal Investigator |  |
| Pinderfields General Hospital | Sarah Buckley |  |  |
| Pinderfields General Hospital | Sarah Boot |  |  |
| Pinderfields General Hospital | Alexandra Metcalfe |  |  |
| Pinderfields General Hospital | Akshay Dwarakanath |  |  |
| Pinderfields General Hospital | James Quinn |  |  |
| Pinderfields General Hospital | Harish Shankar Kumar | Associate PI |  |
| Pinderfields General Hospital | Amy Amy Major |  |  |
| Princess Alexandra Hospital | Peter Russell | Principal Investigator |  |
| Princess Alexandra Hospital | Nikki White |  |  |
| Princess Alexandra Hospital | Patricia Nabayego |  |  |
| Princess Alexandra Hospital | Carol Muir |  |  |
| Princess Alexandra Hospital | Bibi Badal |  |  |
| Princess Alexandra Hospital | Gemma Cook |  |  |
| Princess of Wales Hospital | Ruth Williams | Principal Investigator |  |
| Princess of Wales Hospital | Justyna Mikusek |  |  |
| Princess of Wales Hospital | Lisa Roche |  |  |
| Princess of Wales Hospital | Ellie Davies |  |  |
| Princess Royal Hospital | Nigel Capps | Principal Investigator |  |
| Princess Royal Hospital | Sanal Jose |  |  |
| Princess Royal Hospital | Emily Cale |  |  |
| Princess Royal Hospital | Denise Donaldson |  |  |
| Princess Royal Hospital | Jennifer Nixon |  |  |
| Princess Royal Hospital | Susannah Pajak |  |  |
| Princess Royal Hospital | Sultan Farooq |  |  |
| Princess Royal Hospital | Harmesh Moudgil |  |  |
| Princess Royal Hospital | Koottalai Srinivasan |  |  |
| Princess Royal Hospital | Manoj Marathe |  |  |
| Princess Royal Hospital | Anosha Aslam |  |  |
| Princess Royal Hospital | Jean Nyoni |  |  |
| Princess Royal Hospital | Nawaid Ahmad |  |  |
| Princess Royal Hospital | Elizabeth Mahon |  |  |
| Princess Royal Hospital | James Greenway |  |  |
| Princess Royal Hospital | Wendy Osborne |  |  |
| Queen Alexandra Hospital | Kamran Tariq | Principal Investigator |  |
| Queen Alexandra Hospital | Elizabeth Hawes |  |  |
| Queen Alexandra Hospital | Mary Wands |  |  |
| Queen Alexandra Hospital | Stephanie Hetley |  |  |
| Queen Alexandra Hospital | Kate Burrows |  |  |
| Queen Alexandra Hospital | Laura Wiffen |  |  |
| Queen Alexandra Hospital | Jill Andrews |  |  |
| Queen Alexandra Hospital | Christine Minnis |  |  |
| Queen Alexandra Hospital | Pedro Braga Sardo |  |  |
| Queen Alexandra Hospital | Jingxiu Ouyang |  |  |
| Queen Alexandra Hospital | Eloise Lavington |  |  |
| Queen Elizabeth Hospital | Arun Baral | Principal Investigator |  |
| Queen Elizabeth Hospital | Riyam Al-Sammarraie |  |  |
| Queen Elizabeth Hospital | Evelyn Nadar |  |  |
| Queen Elizabeth Hospital | Zoe Coton |  |  |
| Queen Elizabeth Hospital | Hollie Curgenven |  |  |
| Queen Elizabeth Hospital | Dasa Muzenic |  |  |
| Queen Elizabeth Hospital | Jessica Wong Sun Wai |  |  |
| Queen Elizabeth Hospital | Tha Nyi |  |  |
| Queen Elizabeth Hospital | Sophy Shedwell |  |  |
| Queen Elizabeth Hospital | Kiran Kishore Kondapalakala |  |  |
| Queen Elizabeth Hospital | Rezwana Rahman |  |  |
| Queen Elizabeth Hospital | Edith Njideka Ukaegbu |  |  |
| Queen Elizabeth Hospital | Oluwatoyin Aribike |  |  |
| Queen Elizabeth Hospital | Karmugilan Rajasekar |  |  |
| Queen Elizabeth Hospital | Purnima Ghale |  |  |
| Queen Elizabeth Hospital | Senali Labunahewa |  |  |
| Queen Elizabeth Hospital | Fiona Richardson |  |  |
| Queen Elizabeth Hospital | Anna John Peters |  |  |
| Queen Elizabeth Hospital | Hussein Yousif |  |  |
| Queen Elizabeth Hospital | Aricsa Joshy |  |  |
| Queen Elizabeth Hospital | Grace Hasnip |  |  |
| Queen Elizabeth Hospital | Hayley Webb |  |  |
| Queen Elizabeth Hospital | Felicity Ighofose |  |  |
| Queen Elizabeth Hospital | Tracy Fuller |  |  |
| Queen Elizabeth Hospital | Sharon Steward |  |  |
| Queen Elizabeth Hospital | Elizabeth Newman-Horne |  |  |
| Queen Elizabeth Hospital | Rachel Bocking |  |  |
| Queen Elizabeth Hospital | Uma Stephen Paul |  |  |
| Queen Elizabeth Hospital | Sanjida Akter |  |  |
| Queen Elizabeth Hospital | Mariam Rajani |  |  |
| Queen Elizabeth Hospital | Enamur Rahman |  |  |
| Queen Elizabeth Hospital Birmingham | Dhruv Parekh | Principal Investigator |  |
| Queen Elizabeth Hospital Birmingham | Christopher Mcghee |  |  |
| Queen Elizabeth Hospital Birmingham | Michelle Bates |  |  |
| Queen Elizabeth Hospital Birmingham | Nafeesah Haider |  |  |
| Queen Elizabeth Hospital Birmingham | Emma Burke |  |  |
| Queen Elizabeth Hospital Birmingham | Khushpreet Bhandal |  |  |
| Queen Elizabeth Hospital Birmingham | Muhammad Bilal Khan Niazi | Associate PI |  |
| Queen Elizabeth Hospital Birmingham | Sebastian Lugg |  |  |
| Queen Elizabeth Hospital Birmingham | Shannon Page |  |  |
| Queen Elizabeth Hospital Birmingham | Saleem Chaudhri | Associate PI |  |
| Queen Elizabeth University Hospital | Colin Berry | Principal Investigator |  |
| Queen Elizabeth University Hospital | Karen Pointon |  |  |
| Queen Elizabeth University Hospital | Robert Sykes |  |  |
| Queen Elizabeth University Hospital | Kirsty Mcleish |  |  |
| Queen Elizabeth University Hospital | Linda Taylor |  |  |
| Queen Elizabeth University Hospital | Susan Currie |  |  |
| Queen Elizabeth University Hospital | Susan Speirs |  |  |
| Queen Elizabeth University Hospital | Catherine Clayton |  |  |
| Queen's Hospital, Burton | Uttam Nanda | Principal Investigator |  |
| Queen's Hospital, Burton | Sarah Hathaway-Lees |  |  |
| Queen's Hospital, Burton | Gillian Bell |  |  |
| Queen's Hospital, Burton | Louise Wilcox |  |  |
| Queen's Hospital, Burton | Jess Usher |  |  |
| Queen's Hospital, Burton | Christopher Fewings |  |  |
| Queen's Hospital, Burton | Adrian Mcgrath |  |  |
| Queen's Medical Centre | Stephen Ryder | Principal Investigator |  |
| Queen's Medical Centre | Jan Hallas |  |  |
| Queen's Medical Centre | Jack Squires |  |  |
| Queen's Medical Centre | Emma Kendall |  |  |
| Queen's Medical Centre | Sabrina Prosper |  |  |
| Queen's Medical Centre | Athanasios Nikolaidis |  |  |
| Queen's Medical Centre | Georgia Melia |  |  |
| Queen's Medical Centre | Nicola Benetti |  |  |
| Queen's Medical Centre | Chloe Khurana |  |  |
| Queen's Medical Centre | Marie Quinlan |  |  |
| Queen's Medical Centre | Catherine Dupont |  |  |
| Rotherham District General Hospital | Anil Hormis | Principal Investigator |  |
| Rotherham District General Hospital | Allison Daniels |  |  |
| Rotherham District General Hospital | Cheryl Graham |  |  |
| Rotherham District General Hospital | Julie Ingham |  |  |
| Rotherham District General Hospital | Victoria Maynard |  |  |
| Rotherham District General Hospital | Lisa Zeidan |  |  |
| Rotherham District General Hospital | Louise Weatherley |  |  |
| Royal Albert Edward Infirmary | Imran Aziz | Principal Investigator |  |
| Royal Albert Edward Infirmary | Sarah Liderth |  |  |
| Royal Albert Edward Infirmary | Emma Robinson |  |  |
| Royal Albert Edward Infirmary | Natalia Waddington |  |  |
| Royal Albert Edward Infirmary | Josh Cooper |  |  |
| Royal Albert Edward Infirmary | Jordan Wedlin |  |  |
| Royal Albert Edward Infirmary | Claire Williams |  |  |
| Royal Albert Edward Infirmary | Tracey Taylor |  |  |
| Royal Albert Edward Infirmary | Caroline Tierney |  |  |
| Royal Albert Edward Infirmary | Valerie Parkinson |  |  |
| Royal Alexandra Hospital | Jamie Hornsby | Principal Investigator |  |
| Royal Alexandra Hospital | Alasdair Corfield |  |  |
| Royal Alexandra Hospital | Lynn Abel |  |  |
| Royal Alexandra Hospital | Natalie Rodden |  |  |
| Royal Alexandra Hospital | Nicola Thomson |  |  |
| Royal Alexandra Hospital | Susan Currie |  |  |
| Royal Alexandra Hospital | James Paxton |  |  |
| Royal Berkshire Hospital | Foteini Kavvoura | Principal Investigator |  |
| Royal Berkshire Hospital | Anand Pankhania | Associate PI |  |
| Royal Berkshire Hospital | Julie Sutton |  |  |
| Royal Berkshire Hospital | Cameron Mclaren |  |  |
| Royal Berkshire Hospital | Sabi Gurung Rai |  |  |
| Royal Berkshire Hospital | Jennifer Armistead |  |  |
| Royal Berkshire Hospital | Parminder Bhuie |  |  |
| Royal Berkshire Hospital | Shauna Bartley |  |  |
| Royal Bolton Hospital | Rizwan Ahmed | Principal Investigator |  |
| Royal Bolton Hospital | Scott Latham |  |  |
| Royal Bolton Hospital | Raksha Mistry |  |  |
| Royal Bolton Hospital | Kat Rhead |  |  |
| Royal Bolton Hospital | Emma Mckenna |  |  |
| Royal Bolton Hospital | Aamir Saeed |  |  |
| Royal Bolton Hospital | Jennifer Anderson |  |  |
| Royal Bolton Hospital | Owais Maskati |  |  |
| Royal Bolton Hospital | Karen Lee |  |  |
| Royal Bolton Hospital | Lily Edwards |  |  |
| Royal Bolton Hospital | Jamie Phillips |  |  |
| Royal Bolton Hospital | Amalia Gervasutti |  |  |
| Royal Bolton Hospital | Eve Morrison |  |  |
| Royal Bolton Hospital | Muhammad Nagia |  |  |
| Royal Bolton Hospital | Robert Hull |  |  |
| Royal Cornwall Hospital (Treliske) | Duncan Browne | Principal Investigator |  |
| Royal Cornwall Hospital (Treliske) | Linda Allsop |  |  |
| Royal Cornwall Hospital (Treliske) | Johanna Skewes |  |  |
| Royal Cornwall Hospital (Treliske) | Victoria Edge |  |  |
| Royal Cornwall Hospital (Treliske) | Louise Laity |  |  |
| Royal Cornwall Hospital (Treliske) | Wen Teng Yong |  |  |
| Royal Cornwall Hospital (Treliske) | Amber Wynn |  |  |
| Royal Cornwall Hospital (Treliske) | Tracy Nampa |  |  |
| Royal Cornwall Hospital (Treliske) | Leanne Trethowan |  |  |
| Royal Derby Hospital | Tom Bewick | Principal Investigator |  |
| Royal Derby Hospital | Melanie Hayman |  |  |
| Royal Derby Hospital | Fatima Al-Arrayed | Associate PI |  |
| Royal Derby Hospital | Katie Smith |  |  |
| Royal Derby Hospital | Lucy Boast |  |  |
| Royal Derby Hospital | Nicola Jackson |  |  |
| Royal Derby Hospital | Kate Haggan |  |  |
| Royal Derby Hospital | Coral Smith |  |  |
| Royal Derby Hospital | Jess Usher |  |  |
| Royal Derby Hospital | Suzannah Woodhouse |  |  |
| Royal Derby Hospital | Leanne Lacey |  |  |
| Royal Derby Hospital | Safeyah Ahmed |  |  |
| Royal Free Hospital | Swapna Mandal | Principal Investigator |  |
| Royal Free Hospital | Amar Shah |  |  |
| Royal Free Hospital | Katia Florman | Associate PI |  |
| Royal Free Hospital | Aarti Nandani |  |  |
| Royal Infirmary of Edinburgh | Alasdair Grey | Principal Investigator |  |
| Royal Infirmary of Edinburgh | Polly Black |  |  |
| Royal Infirmary of Edinburgh | Scott Morrison |  |  |
| Royal Infirmary of Edinburgh | David Henshall |  |  |
| Royal Infirmary of Edinburgh | Rachel O'Brien |  |  |
| Royal Infirmary of Edinburgh | Emily Godden |  |  |
| Royal Infirmary of Edinburgh | James Dear |  |  |
| Royal Infirmary of Edinburgh | Julia Grahamslaw |  |  |
| Royal Infirmary of Edinburgh | Fiona McCurrach |  |  |
| Royal Infirmary of Edinburgh | Alison Williams |  |  |
| Royal Infirmary of Edinburgh | Sanjita Brito Mutunayagam | Associate PI |  |
| Royal Infirmary of Edinburgh | Jack Cafferkey |  |  |
| Royal Infirmary of Edinburgh | Nicola Freeman |  |  |
| Royal Infirmary of Edinburgh | Alison Grant |  |  |
| Royal Infirmary of Edinburgh | Anastasia Levynska |  |  |
| Royal Infirmary of Edinburgh | Liza Greenhalgh |  |  |
| Royal Infirmary of Edinburgh | Sven Nelson |  |  |
| Royal Infirmary of Edinburgh | Louise Sharp |  |  |
| Royal Infirmary of Edinburgh | Anne Saunderson |  |  |
| Royal Liverpool University Hospital | Paul Hine | Principal Investigator |  |
| Royal Liverpool University Hospital | Sharon Glynn |  |  |
| Royal Liverpool University Hospital | Deborah Scanlon |  |  |
| Royal Liverpool University Hospital | Eleanor Tyaylor-Barr |  |  |
| Royal Liverpool University Hospital | Claire Small |  |  |
| Royal Liverpool University Hospital | Flora Malein |  |  |
| Royal Liverpool University Hospital | Mary Brodsky |  |  |
| Royal Liverpool University Hospital | Marcella Vaselli |  |  |
| Royal Liverpool University Hospital | Alvyda Gureviciute |  |  |
| Royal Liverpool University Hospital | Debbie Heath |  |  |
| Royal Liverpool University Hospital | Nathalie Nicholas |  |  |
| Royal Liverpool University Hospital | Stacy Todd |  |  |
| Royal Liverpool University Hospital | Tomos Evans |  |  |
| Royal Liverpool University Hospital | Pinky Thu-Ta |  |  |
| Royal Liverpool University Hospital | Jennifer Tempany |  |  |
| Royal Liverpool University Hospital | Alexander Morgan |  |  |
| Royal Liverpool University Hospital | Shane D'souza |  |  |
| Royal Liverpool University Hospital | Lydia Hawker |  |  |
| Royal Liverpool University Hospital | Abolaji Atomode | Associate PI |  |
| Royal Liverpool University Hospital | Melanie Hamilton |  |  |
| Royal Liverpool University Hospital | Jack Goodall |  |  |
| Royal Liverpool University Hospital | Megan Howard |  |  |
| Royal Liverpool University Hospital | Emma Richardson |  |  |
| Royal Liverpool University Hospital | Victoria Simpson |  |  |
| Royal London Hospital | Heinke Kunst | Principal Investigator |  |
| Royal London Hospital | Mylah Ramirez |  |  |
| Royal London Hospital | Ananna Rahman |  |  |
| Royal London Hospital | Catherine Heckman |  |  |
| Royal London Hospital | Malcolm Avari |  |  |
| Royal London Hospital | William Hann |  |  |
| Royal London Hospital | Neena Patel |  |  |
| Royal London Hospital | Alexandra Lawrence |  |  |
| Royal London Hospital | Antonietta Rocca Fonseca |  |  |
| Royal London Hospital | Martin Yardley |  |  |
| Royal London Hospital | Lioniza Mayola |  |  |
| Royal London Hospital | Sophie Masterson |  |  |
| Royal London Hospital | Aarash Saleh |  |  |
| Royal Papworth Hospital | Michael Davies | Principal Investigator |  |
| Royal Papworth Hospital | Lucie Garner |  |  |
| Royal Papworth Hospital | Joseph Newman |  |  |
| Royal Papworth Hospital | Kitty Paques |  |  |
| Royal Papworth Hospital | Iryna Boubriak | Associate PI |  |
| Royal Papworth Hospital | Mohamed Ben M'barek |  |  |
| Royal Papworth Hospital | Alaa Alsaaty | Associate PI |  |
| Royal Papworth Hospital | Keshini Kulathevanayagam | Associate PI |  |
| Royal Papworth Hospital | Frederick Jarvis | Associate PI |  |
| Royal Preston Hospital | Sharada Gudur | Principal Investigator |  |
| Royal Preston Hospital | Amanda Alty |  |  |
| Royal Preston Hospital | Sandra Sowden |  |  |
| Royal Preston Hospital | Mark Verlander |  |  |
| Royal Preston Hospital | Toni Sutcliffe-Whyte |  |  |
| Royal Preston Hospital | Ailsa Watt |  |  |
| Royal Preston Hospital | Janet Mills |  |  |
| Royal Preston Hospital | Willy Choon Kon Yune | Associate PI |  |
| Royal Preston Hospital | Saba Hamad |  |  |
| Royal Preston Hospital | Saleel Punnilath Abdulsamad |  |  |
| Royal Preston Hospital | Imran Haidry |  |  |
| Royal Shrewsbury Hospital | Nigel Capps | Principal Investigator |  |
| Royal Shrewsbury Hospital | Sanal Jose |  |  |
| Royal Shrewsbury Hospital | Angela Yeomans |  |  |
| Royal Shrewsbury Hospital | Jo Stickley |  |  |
| Royal Shrewsbury Hospital | Alison Stephens |  |  |
| Royal Shrewsbury Hospital | Alexander Moorcroft |  |  |
| Royal Shrewsbury Hospital | Richard Heinink |  |  |
| Royal Shrewsbury Hospital | Nicola Rowe |  |  |
| Royal Shrewsbury Hospital | Laura Price |  |  |
| Royal Shrewsbury Hospital | Susannah Pajak |  |  |
| Royal Shrewsbury Hospital | Nihal Mehra |  |  |
| Royal Shrewsbury Hospital | Raja Rajan Anandavelu |  |  |
| Royal Shrewsbury Hospital | Graham Heyes |  |  |
| Royal Shrewsbury Hospital | Jemma Pearson |  |  |
| Royal Shrewsbury Hospital | Olivia Walsh |  |  |
| Royal Surrey County Hospital | Kath Mccullough | Principal Investigator |  |
| Royal Surrey County Hospital | Charles Piercy |  |  |
| Royal Surrey County Hospital | Esther Tarr |  |  |
| Royal Surrey County Hospital | Armorel Salberg |  |  |
| Royal Surrey County Hospital | Sarah Stone |  |  |
| Royal Surrey County Hospital | Irving Cens Mayangao |  |  |
| Royal Surrey County Hospital | John De Vos |  |  |
| Royal Surrey County Hospital | Kimberley Lucini |  |  |
| Royal Surrey County Hospital | Sinead Donlon |  |  |
| Royal Surrey County Hospital | Hyacinth-John Abu |  |  |
| Royal Surrey County Hospital | Eleanor Smith |  |  |
| Royal Surrey County Hospital | Susan Bowles |  |  |
| Royal Surrey County Hospital | Joanna Smith |  |  |
| Royal United Hospital | Jay Suntharalingam | Principal Investigator |  |
| Royal United Hospital | Katie White |  |  |
| Royal United Hospital | Alison Kirby |  |  |
| Royal United Hospital | Carrie Demetriou |  |  |
| Royal United Hospital | Lidia Ramos |  |  |
| Royal United Hospital | Annette Seatter |  |  |
| Royal United Hospital | Helen Burton |  |  |
| Royal United Hospital | Tonia Clark |  |  |
| Royal United Hospital | Margaret Macmillan |  |  |
| Royal United Hospital | Lauren Pearce |  |  |
| Royal United Hospital | Telma Costa |  |  |
| Royal United Hospital | Sharon Sturney |  |  |
| Royal United Hospital | Ioana Fodor |  |  |
| Royal United Hospital | Clare Marchand |  |  |
| Royal United Hospital | Robert Mackenzie Ross |  |  |
| Royal United Hospital | Rebecca Mason |  |  |
| Royal United Hospital | Vidan Masani |  |  |
| Royal United Hospital | Tom Hartley |  |  |
| Royal United Hospital | Chloe Parrish |  |  |
| Royal United Hospital | Helen Mcdill |  |  |
| Royal United Hospital | Jack Evans |  |  |
| Royal United Hospital | James Harper |  |  |
| Royal United Hospital | Jonathan Noble |  |  |
| Royal United Hospital | Zandile Maseko |  |  |
| Royal Victoria Hospital | Danny McAuley | Principal Investigator |  |
| Royal Victoria Hospital | Roisin Stone |  |  |
| Royal Victoria Hospital | Danielle Dawson |  |  |
| Royal Victoria Hospital | Jonathan Stewart | Associate PI |  |
| Royal Victoria Hospital | Brian Wells |  |  |
| Royal Victoria Hospital | Karan Smyth |  |  |
| Royal Victoria Hospital | Alexandra Usher-Rea |  |  |
| Royal Victoria Hospital | Siobhan Moor |  |  |
| Royal Victoria Hospital | Samantha Murphy |  |  |
| Salford Royal Hospital | Darren Green | Principal Investigator |  |
| Salford Royal Hospital | Bethan Charles |  |  |
| Salford Royal Hospital | Hannah Merrill | Associate PI |  |
| Salford Royal Hospital | Diane Lomas |  |  |
| Salford Royal Hospital | Karen Knowles |  |  |
| Salford Royal Hospital | Reece Doonan |  |  |
| Salford Royal Hospital | Tracy Marsden |  |  |
| Salford Royal Hospital | Matthew Collis |  |  |
| Salford Royal Hospital | Natalie Thomas |  |  |
| Salford Royal Hospital | Olivia Wickens |  |  |
| Salford Royal Hospital | Diana Chiu |  |  |
| Salford Royal Hospital | Melanie Taylor |  |  |
| Salford Royal Hospital | Alice Harvey |  |  |
| Salford Royal Hospital | Stephanie Lee |  |  |
| Salford Royal Hospital | Vicky Thomas |  |  |
| Salford Royal Hospital | Jack Hodd |  |  |
| Salford Royal Hospital | Danielle Mclaughlan |  |  |
| Salford Royal Hospital | Sean Knight |  |  |
| Salisbury District Hospital | Jonathan Cullis | Principal Investigator |  |
| Salisbury District Hospital | Catherine Reed |  |  |
| Salisbury District Hospital | Beena Eapen |  |  |
| Salisbury District Hospital | Lenka Cambalova |  |  |
| Salisbury District Hospital | Kerryn Hudson |  |  |
| Salisbury District Hospital | Louise Gamble |  |  |
| Salisbury District Hospital | James Milnthorpe |  |  |
| Salisbury District Hospital | Wadzanai Matimba-Mupaya |  |  |
| Salisbury District Hospital | Holly Morgan |  |  |
| Salisbury District Hospital | Lijo Joy |  |  |
| Salisbury District Hospital | Kate Ames |  |  |
| Salisbury District Hospital | Abby Rand |  |  |
| Salisbury District Hospital | Alpha Anthony |  |  |
| Salisbury District Hospital | Victoria King |  |  |
| Salisbury District Hospital | Sophia Strong-Sheldrake |  |  |
| Salisbury District Hospital | Rohan Mehta |  |  |
| Salisbury District Hospital | Catherine Thompson |  |  |
| Salisbury District Hospital | Sian Evans |  |  |
| Salisbury District Hospital | Susil Isaac |  |  |
| Scunthorpe General Hospital | Liaquat Ali | Principal Investigator |  |
| Scunthorpe General Hospital | Dorothy Hutchinson |  |  |
| Scunthorpe General Hospital | Sandra Pearson |  |  |
| Scunthorpe General Hospital | Kirsty Nauyokas |  |  |
| Sheffield Teaching Hospitals | Roger Thompson | Principal Investigator |  |
| Sheffield Teaching Hospitals | Claire Jarman |  |  |
| Sheffield Teaching Hospitals | Rosemary Kirk |  |  |
| Sheffield Teaching Hospitals | Joby Cole |  |  |
| Sheffield Teaching Hospitals | Phillip Wade |  |  |
| Sheffield Teaching Hospitals | Alison Lye |  |  |
| Sheffield Teaching Hospitals | Helen Newell |  |  |
| Sheffield Teaching Hospitals | Kate Harrington |  |  |
| Sheffield Teaching Hospitals | Sara Jane Walker |  |  |
| Sheffield Teaching Hospitals | Lynne Smart |  |  |
| Sheffield Teaching Hospitals | Lorenza Onyinyechi Nwafor |  |  |
| Sheffield Teaching Hospitals | Sharon Megson |  |  |
| Sheffield Teaching Hospitals | Tom Newman |  |  |
| Sheffield Teaching Hospitals | Megan Plowright | Associate PI |  |
| Sheffield Teaching Hospitals | Jessica Mcneill |  |  |
| Sheffield Teaching Hospitals | Mariam Ilyas |  |  |
| Sheffield Teaching Hospitals | Falastina Ahmed |  |  |
| Singleton Hospital | Ian Blyth | Principal Investigator |  |
| Singleton Hospital | Tabitha Rees |  |  |
| Singleton Hospital | Caradog Thomas |  |  |
| Singleton Hospital | Rachel Harford |  |  |
| Singleton Hospital | Carl Murphy |  |  |
| Singleton Hospital | Amanda Cook |  |  |
| Singleton Hospital | Elaine Brinkworth |  |  |
| Singleton Hospital | Caroline Davies |  |  |
| Southmead Hospital | Ed Moran | Principal Investigator |  |
| Southmead Hospital | Rachel Williams |  |  |
| Southmead Hospital | Kirstie Bradburn |  |  |
| Southmead Hospital | Holly Thomas |  |  |
| Southmead Hospital | Grace Okoro |  |  |
| Southmead Hospital | Elizabeth Barnett |  |  |
| Southmead Hospital | Beverley Faulkner |  |  |
| Southmead Hospital | Louise Solomon |  |  |
| Southmead Hospital | Alyssa D'agostino |  |  |
| Southmead Hospital | Catherine Floutier |  |  |
| Southmead Hospital | James Thomas |  |  |
| Southmead Hospital | Maisie Borrill |  |  |
| Southmead Hospital | Jennifer Tomlins | Associate PI |  |
| St Helier Hospital | Rebecca Macfarlane | Principal Investigator |  |
| St Helier Hospital | Victoria Taylor | Associate PI |  |
| St Helier Hospital | Emese Balogh |  |  |
| St Helier Hospital | Lisa Evans |  |  |
| St Helier Hospital | Annie Mathew |  |  |
| St Helier Hospital | Nicole Blanco |  |  |
| St Helier Hospital | Manuel Rebolledo Romero |  |  |
| St Helier Hospital | Grace Blows |  |  |
| St Helier Hospital | Amy Matthews |  |  |
| St James's University Hospital | Kyra Holliday | Principal Investigator |  |
| St James's University Hospital | Amy Humphries |  |  |
| St James's University Hospital | Jude Clarke |  |  |
| St James's University Hospital | Jodie Glossop |  |  |
| St James's University Hospital | Lucy Hall |  |  |
| St James's University Hospital | Gwendolyn Saalmink |  |  |
| St James's University Hospital | Elaine Wade |  |  |
| St James's University Hospital | Sharon James |  |  |
| St James's University Hospital | Andrew Ashworth |  |  |
| St James's University Hospital | Asad Ali |  |  |
| St James's University Hospital | Clair Favager |  |  |
| St John's Hospital | Stephen Lynch | Principal Investigator |  |
| St John's Hospital | Rachel O'brien |  |  |
| St John's Hospital | Stephen Lynch |  |  |
| St John's Hospital | Claire Cheyne |  |  |
| St John's Hospital | Liam Turnbull |  |  |
| St Mary's Hospital | Onn Min Kon | Principal Investigator |  |
| St Mary's Hospital | Nelisa Poshai |  |  |
| St Mary's Hospital | Joan Nanan |  |  |
| St Mary's Hospital | Amal Jama |  |  |
| St Mary's Hospital | Alexandra Cann |  |  |
| St Mary's Hospital | Chloë Wood |  |  |
| St Mary's Hospital | Jisha Mathew |  |  |
| St Mary's Hospital | Tamanah Fayzan |  |  |
| St Mary's Hospital | Hannah Rafferty |  |  |
| St Mary's Hospital | Beatriz Tomas-Cordero |  |  |
| St Mary's Hospital | Emma Sidebotham |  |  |
| St Mary's Hospital | Jessica Tuff |  |  |
| St Mary's Hospital | Simran Johal |  |  |
| St Mary's Hospital | Mohammed Rahman |  |  |
| St Mary's Hospital | Mark Elliott |  |  |
| St Mary's Hospital | Adedeji Ogunlana |  |  |
| St Mary's Hospital | Zayneb Al-Saadi |  |  |
| St Mary's Hospital | Clive Matthews |  |  |
| St Mary's Hospital | Marcelino Molina |  |  |
| St Mary's Hospital | Madeline Fox |  |  |
| St Thomas's Hospital | Marlies Ostermann | Principal Investigator |  |
| St Thomas's Hospital | Noelia Amutio Martin |  |  |
| St Thomas's Hospital | Helen Kerslake |  |  |
| St Thomas's Hospital | Kiki Burns |  |  |
| St Thomas's Hospital | Murphy Magtoto |  |  |
| St Thomas's Hospital | Megan O Toole |  |  |
| St Thomas's Hospital | Charlotte Richards |  |  |
| St Thomas's Hospital | Jo Salkeld |  |  |
| St Thomas's Hospital | Sarah Hammett |  |  |
| St Thomas's Hospital | Yee Wing Maxine Ng |  |  |
| St Thomas's Hospital | Lauren Martinez |  |  |
| St Thomas's Hospital | Laura Aslett |  |  |
| St Thomas's Hospital | Suhail Aslam |  |  |
| St Thomas's Hospital | Nuttha Lumlertgul | Associate PI |  |
| St Thomas's Hospital | Laura Aguilar Jimenez |  |  |
| St Thomas's Hospital | Sahnaj Sultana |  |  |
| St Thomas's Hospital | Pamela De Los Santos Dominguez |  |  |
| St Thomas's Hospital | Alison Davies |  |  |
| St Thomas's Hospital | Junelyn Gozo |  |  |
| St Thomas's Hospital | Sarah Betts |  |  |
| St Thomas's Hospital | Teona Serafimova |  |  |
| St Thomas's Hospital | Tanveer Bawa |  |  |
| St Thomas's Hospital | Niamh Spence |  |  |
| St Thomas's Hospital | Movin Abeywickrema |  |  |
| St Thomas's Hospital | Aisling Brown |  |  |
| St Thomas's Hospital | Alice Packham |  |  |
| St Thomas's Hospital | Teresa Crowley |  |  |
| St Thomas's Hospital | Irene Cantante |  |  |
| St Thomas's Hospital | Ramona Lungu |  |  |
| St Thomas's Hospital | Krishna Satchithananthasivam |  |  |
| St Thomas's Hospital | Cherry Paice |  |  |
| St Thomas's Hospital | Carlo D'aloia |  |  |
| St Thomas's Hospital | Anbhu Balasubramanian |  |  |
| St Thomas's Hospital | Caitlin Mcgreevy |  |  |
| St Thomas's Hospital | Emily Palmer |  |  |
| St Thomas's Hospital | Denise Prizis |  |  |
| St Thomas's Hospital | Shania Lorenz |  |  |
| St Thomas's Hospital | Sherin John |  |  |
| St Thomas's Hospital | Maria Mercado |  |  |
| St Thomas's Hospital | Naswiib Kasozi |  |  |
| St Thomas's Hospital | Andrzej Szumko |  |  |
| St Thomas's Hospital | Litty Jose |  |  |
| Stoke Mandeville Hospital | Mitra Shahidi | Principal Investigator |  |
| Stoke Mandeville Hospital | Katarina Manso |  |  |
| Stoke Mandeville Hospital | Hsu Myat Noe |  |  |
| Stoke Mandeville Hospital | Ben Marks |  |  |
| Stoke Mandeville Hospital | Siobhan Gettings |  |  |
| Stoke Mandeville Hospital | Judith Abrams |  |  |
| Stoke Mandeville Hospital | Mirella Corredera |  |  |
| Stoke Mandeville Hospital | Paula Jenkins |  |  |
| Stoke Mandeville Hospital | Bethan Davies |  |  |
| Stoke Mandeville Hospital | Alice Ngumo |  |  |
| Stoke Mandeville Hospital | Rossana Mancinelli |  |  |
| University College Hospital | Michael Marks | Principal Investigator |  |
| University College Hospital | Eiko Tomokiyo |  |  |
| University College Hospital | Harriet Sorrell |  |  |
| University College Hospital | Antonette Andrews |  |  |
| University College Hospital | Sarah Logan |  |  |
| University Hospital Aintree | Paul Albert | Principal Investigator |  |
| University Hospital Aintree | Sharon Glynn |  |  |
| University Hospital Aintree | Leigh Pauls |  |  |
| University Hospital Aintree | Anna Rowe |  |  |
| University Hospital Aintree | Nathalie Nicholas |  |  |
| University Hospital Aintree | Debbie Heath |  |  |
| University Hospital Aintree | Paul Hine |  |  |
| University Hospital Aintree | Mary Brodsky |  |  |
| University Hospital Aintree | Pinky Thu-Ta |  |  |
| University Hospital Aintree | Alice Withington |  |  |
| University Hospital Aintree | Hon-Ting Indy Wai |  |  |
| University Hospital Aintree | Paul-Peter Merron |  |  |
| University Hospital Aintree | John Doherty | Associate PI |  |
| University Hospital Aintree | Dhruv Khanna |  |  |
| University Hospital Aintree | Jennifer Miller |  |  |
| University Hospital Hairmyres | Manish Patel | Principal Investigator |  |
| University Hospital Hairmyres | Lynn Glass |  |  |
| University Hospital Hairmyres | Emma Lee |  |  |
| University Hospital Hairmyres | Margaret Mcfadden |  |  |
| University Hospital Hairmyres | Pauleen Grant |  |  |
| University Hospital Hairmyres | Tracy Baird |  |  |
| University Hospital Hairmyres | Rajni Tejwani |  |  |
| University Hospital Hairmyres | Claire Beith |  |  |
| University Hospital Hairmyres | Elizabeth Lennnon |  |  |
| University Hospital Hairmyres | Louise Clark |  |  |
| University Hospital Hairmyres | Louise Jamieson |  |  |
| University Hospital of North Durham | Fatima Khalil | Principal Investigator |  |
| University Hospital of North Durham | Andrea Kay |  |  |
| University Hospital of North Durham | Mark Birt |  |  |
| University Hospital of North Durham | Kishor Lekhak | Associate PI |  |
| University Hospital of North Durham | Noreen Kingston |  |  |
| University Hospital of North Durham | Kathryn Potts |  |  |
| University Hospital of North Durham | Victoria Allinson |  |  |
| University Hospital of North Tees | Kevin Conroy | Principal Investigator |  |
| University Hospital of North Tees | Lorna Shepherd |  |  |
| University Hospital of North Tees | Lynda Poole |  |  |
| University Hospital of North Tees | Julie Quigley |  |  |
| University Hospital of North Tees | Debbie Wilson |  |  |
| University Hospital of North Tees | Simon Sinclair |  |  |
| University Hospital of North Tees | Claire Riley |  |  |
| University Hospital of North Tees | Jill Skelton |  |  |
| University Hospital of North Tees | Alex Ramshaw |  |  |
| University Hospital of North Tees | Fe Marie Hernandez |  |  |
| University Hospital of North Tees | Maria Weetman |  |  |
| University Hospital of North Tees | Sarah Purvis |  |  |
| University Hospital of North Tees | Jake Mcgee |  |  |
| University Hospital of North Tees | Alison Chilvers |  |  |
| University Hospital of North Tees | Elaine Siddle |  |  |
| University Hospital of North Tees | Luke Ventress |  |  |
| University Hospital of North Tees | Alexandra Tonks |  |  |
| University Hospital of North Tees | Graham Miller |  |  |
| University Hospital of North Tees | Benjamin Prudon |  |  |
| University Hospital of North Tees | Gillian Wallace |  |  |
| University Hospital of North Tees | Osama Alhabsha |  |  |
| University Hospital of North Tees | Barbara Campbell |  |  |
| University Hospital of North Tees | Sze Hwei Ooi |  |  |
| University Hospital of North Tees | Mohamed Ali |  |  |
| University Hospital of North Tees | Thamani Mazhani |  |  |
| University Hospital of North Tees | Lessica Miel Magnaye |  |  |
| University Hospital of North Tees | Alice Stanton |  |  |
| University Hospital of Wales | Jonathan Underwood | Principal Investigator |  |
| University Hospital of Wales | Teriann Evans |  |  |
| University Hospital of Wales | Oliver Smart |  |  |
| University Hospital of Wales | Sharon Frayling |  |  |
| University Hospital of Wales | Matthew Haynes |  |  |
| University Hospital of Wales | Catherine Oliver |  |  |
| University Hospital of Wales | Jonathan Charles |  |  |
| University Hospital of Wales | Lauren Broad |  |  |
| University Hospital of Wales | Katie Pink |  |  |
| University Hospital of Wales | Laura Jones |  |  |
| University Hospital of Wales | James Lloyd |  |  |
| University Hospital of Wales | Adam Heavens |  |  |
| University Hospital of Wales | Bethan Lloyd |  |  |
| University Hospital of Wales | Alexander Williams |  |  |
| University Hospital of Wales | Jaydee Pangan |  |  |
| University Hospital of Wales | Rhys Thomas |  |  |
| Victoria Hospital | Patrick Liu | Principal Investigator |  |
| Victoria Hospital | Susan Fowler |  |  |
| Victoria Hospital | Patricia Cochrane |  |  |
| Victoria Hospital | Sandra Pirie |  |  |
| Victoria Hospital | Maria Simpson |  |  |
| Victoria Hospital | Janine Ramsay |  |  |
| Victoria Hospital | Simon Finch |  |  |
| Watford General Hospital | Rama Vancheeswaran | Principal Investigator |  |
| Watford General Hospital | Deepali Oza |  |  |
| Watford General Hospital | Siobhain Carmody |  |  |
| Watford General Hospital | Chiara Ellis |  |  |
| Watford General Hospital | Lynn Denham |  |  |
| Watford General Hospital | Saul Sundayi |  |  |
| Watford General Hospital | Helen Newman |  |  |
| West Cumberland Hospital | Clive Graham | Principal Investigator |  |
| West Cumberland Hospital | Rachel Mutch |  |  |
| West Cumberland Hospital | Hannah Craig |  |  |
| West Cumberland Hospital | Rosemary Harper |  |  |
| Western General Hospital | Oliver Koch | Principal Investigator |  |
| Western General Hospital | Susie Ferguson |  |  |
| Western General Hospital | Amy Shepherd |  |  |
| Western General Hospital | Sheila Morris |  |  |
| Western General Hospital | Morgan Evans |  |  |
| Western General Hospital | Callum Mutch |  |  |
| Western General Hospital | Alexander Christides |  |  |
| Western General Hospital | Sanjita Brito Mutunayagam |  |  |
| Western General Hospital | Yong Yong Tew |  |  |
| Western General Hospital | Min Ke |  |  |
| Western General Hospital | Iain Page |  |  |
| Western General Hospital | Sarah Clifford |  |  |
| Weston General Hospital | Emma Stratton | Principal Investigator |  |
| Weston General Hospital | Robert Duncan | Associate PI |  |
| Weston General Hospital | Susan Wilkinson |  |  |
| Weston General Hospital | Kristina Owens |  |  |
| Weston General Hospital | Lindy Murray |  |  |
| Weston General Hospital | Debra Chatterton |  |  |
| Weston General Hospital | Michael Haley |  |  |
| Weston General Hospital | Edel Robbins |  |  |
| Weston General Hospital | Abdul Bhat |  |  |
| Weston General Hospital | Robert Duncan |  |  |
| Weston General Hospital | Somto Eruchie |  |  |
| Weston General Hospital | Alice Lagnado |  |  |
| Weston General Hospital | Abigail Cannon |  |  |
| Whipps Cross Hospital | Heinke Kunst | Principal Investigator |  |
| Whipps Cross Hospital | Neena Patel |  |  |
| Whipps Cross Hospital | Thomas Swaine |  |  |
| Whipps Cross Hospital | Mylah Ramirez |  |  |
| Whipps Cross Hospital | Ananna Rahman |  |  |
| Whipps Cross Hospital | Catherine Heckman |  |  |
| Whipps Cross Hospital | Malcolm Avari |  |  |
| Whipps Cross Hospital | William Hann |  |  |
| Whipps Cross Hospital | Philippa Kaina |  |  |
| Wishaw General Hospital | Manish Patel | Principal Investigator |  |
| Wishaw General Hospital | Emma Lee |  |  |
| Wishaw General Hospital | Lynn Glass |  |  |
| Wishaw General Hospital | Margaret Mcfadden |  |  |
| Wishaw General Hospital | Tracy Baird |  |  |
| Wishaw General Hospital | Suzanne Clements |  |  |
| Wishaw General Hospital | Clare Brown |  |  |
| Wishaw General Hospital | Andrew Smith |  |  |
| Wishaw General Hospital | Lucia Di Mascio |  |  |
| Wythenshawe Hospital | Sheila Ramjug | Principal Investigator |  |
| Wythenshawe Hospital | Hannah Fabian |  |  |
| Wythenshawe Hospital | Angela Chrisopoulou |  |  |
| Wythenshawe Hospital | Luke Ward |  |  |
| Wythenshawe Hospital | Vivienne Benson |  |  |
| Wythenshawe Hospital | Kristina Stewart |  |  |
| Wythenshawe Hospital | Heather Mcmullen |  |  |
| Wythenshawe Hospital | Jonathan Patachako |  |  |
| Wythenshawe Hospital | Thomas Scoones |  |  |
| Wythenshawe Hospital | Geoffrey Lawrence |  |  |
| Yeovil District Hospital | Andrew Broadley | Principal Investigator |  |
| Yeovil District Hospital | Sarah Board |  |  |
| Yeovil District Hospital | Nicky Marks |  |  |
| Yeovil District Hospital | Kate Beesley |  |  |
| Yeovil District Hospital | Clare Buckley |  |  |
| Yeovil District Hospital | Lucy Pippard |  |  |
| Yeovil District Hospital | Sherly Pallipparambil Antony |  |  |
| Yeovil District Hospital | Amer Rasheed |  |  |
| Yeovil District Hospital | Alison Lewis |  |  |
| Yeovil District Hospital | Diane Wood |  |  |
| Ysbyty Glan Clwyd | Daniel Menzies | Principal Investigator |  |
| Ysbyty Glan Clwyd | Joanne Lewis |  |  |
| Ysbyty Glan Clwyd | Rachel Manley |  |  |
| Ysbyty Glan Clwyd | Tracy Roberts |  |  |
| Ysbyty Glan Clwyd | Ahmed Abou-Haggar |  |  |
| Ysbyty Glan Clwyd | Gwyneth Davies |  |  |
| Ysbyty Glan Clwyd | Stephanie Rees |  |  |
| Ysbyty Glan Clwyd | Riyam Al-Sammarraie |  |  |
| Ysbyty Glan Clwyd | Alexander Van Loggerenberg |  |  |
| Ysbyty Glan Clwyd | Callum Mackay |  |  |
| Ysbyty Glan Clwyd | Saad Qutab |  |  |
| Ysbyty Glan Clwyd | Elkhalefa Marjan |  |  |
| Ysbyty Glan Clwyd | Annette Bolger |  |  |
| Ysbyty Gwynedd | Christian Subbe | Principal Investigator |  |
| Ysbyty Gwynedd | Ellen Knights |  |  |
| Ysbyty Gwynedd | Julia Roberts |  |  |
| Ysbyty Gwynedd | Alice Thomas |  |  |
| Ysbyty Gwynedd | Caryl Butterworth |  |  |
| Ysbyty Gwynedd | Keelie Richardson |  |  |
| Ysbyty Gwynedd | Sharon Evans |  |  |
| Ysbyty Gwynedd | Jeannie Bishop |  |  |
| Ysbyty Gwynedd | Wendy Scrase |  |  |
| Ysbyty Gwynedd | Gareth Jones |  |  |
| Ysbyty Gwynedd | Emma Stenson |  |  |
| Ysbyty Gwynedd | Nabeel Abdallah |  |  |
| Ysbyty Gwynedd | Sulaiman Rawashdeh |  |  |
| Ysbyty Gwynedd | Sreejith Venugopal |  |  |
| Ysbyty Gwynedd | Michael Habashi |  |  |
